# Mechanism of autophagy induced by activation of the AMPK/ERK/mTOR signaling pathway after TRIM22-mediated DENV-2 infection of HUVECs

**DOI:** 10.1186/s12985-022-01932-w

**Published:** 2022-12-31

**Authors:** Ning Wu, Xiaoqin Gou, Pan Hu, Yao Chen, Jinzhong Ji, Yuanying Wang, Li Zuo

**Affiliations:** 1 Chemistry and Biochemistry Laboratory, Guizhou Medical University, Guiyang, China; 2 Department of Immunology, Guizhou Medical University, Guiyang, China

**Keywords:** DENV-2, Autophagy, AMPK/ERK/mTOR signaling pathway, TRIM22

## Abstract

**Background:**

Dengue virus type 2 (DENV-2) was used to infect primary human umbilical vein endothelial cells (HUVECs) to examine autophagy induced by activation of the adenosine monophosphate-activated protein kinase (AMPK)/extracellular signal-regulated kinase (ERK)/mammalian target of rapamycin (mTOR) signaling pathway following tripartite motif-containing 22 (TRIM22)-mediated DENV-2 infection to further reveal the underlying pathogenic mechanism of DENV-2 infection.

**Methods:**

Quantitative real-time polymerase chain reaction (qRT-PCR) was used to screen putative interference targets of TRIM22 and determine the knockdown efficiency. The effect of TRIM22 knockdown on HUVEC proliferation was determined using the CCK8 assay. Following TRIM22 knockdown, transmission electron microscopy (TEM) was used to determine the ultrastructure of HUVEC autophagosomes and expression of HUVEC autophagy and AMPK pathway-related genes were measured by qRT-PCR. Moreover, HUVEC autophagy and AMPK pathway-related protein expression levels were determined by western blot analysis. Cell cycle and apoptosis were assessed by flow cytometry (FCM) and the autophagosome structure of the HUVECs was observed by TEM.

**Results:**

Western blot results indicated that TRIM22 protein expression levels increased significantly 36 h after DENV-2 infection, which was consistent with the proteomics prediction. The CCK8 assay revealed that HUVEC proliferation was reduced following TRIM22 knockdown (*P* < 0.001). The TEM results indicated that HUVEC autolysosomes increased and autophagy was inhibited after TRIM22 knockdown. The qRT-PCR results revealed that after TRIM22 knockdown, the expression levels of antithymocyte globulin 7 (ATG7), antithymocyte globulin 5 (ATG5), Beclin1, ERK, and mTOR genes decreased (*P* < 0.01); however, the expression of AMPK genes (*P* < 0.05) and P62 genes (*P* < 0.001) increased. FCM revealed that following TRIM22 knockdown, the percentage of HUVECs in the G2 phase increased (*P* < 0.001) along with cell apoptosis. The effect of TRIM22 overexpression on HUVEC autophagy induced by DENV-2 infection and AMPK pathways decreased after adding an autophagy inhibitor.

**Conclusions:**

In HUVECs, TRIM22 protein positively regulates autophagy and may affect autophagy through the AMPK/ERK/mTOR signaling pathway. Autophagy is induced by activation of the AMPK/ERK/mTOR signaling pathway following TRIM22-mediated DENV-2 infection of HUVECs.

**Supplementary Information:**

The online version contains supplementary material available at 10.1186/s12985-022-01932-w.

## Background

Dengue virus (DENV) is a positive-sense, single-stranded RNA virus with a diameter of 50 nm. It is approximately 11 kd long and belongs to the *Flavivirus* genus, Flaviviridae. Humans are generally susceptible to DENV and are a natural DENV host [1]. According to statistics, ~ 3.6 billion people are at risk worldwide [2, 3] and more than 21,000 people die each year. Thus, DENV infection has become a major public health problem of global concern. DENV has four serotypes, i.e., DENV1–4, of which DENV-2 is the most widely transmitted [4]. The main spreading process of DENV is relatively simple. After a female mosquito feeds on the blood of a DENV patient, DENV multiplies in the mosquito body and spreads to susceptible individuals through mosquito bites [5]. DENV infection causes several self-limiting febrile diseases including dengue fever (DF), dengue hemorrhagic fever (DHF), and dengue shock syndrome (DSS). DHF and DSS are fatal syndromes with clinical manifestations of increased vascular permeability and plasma leakage [6], and further result in multiple organ damage and circulatory system failure, which endangers life [7–9]. Pathogenesis is associated with the dysfunction of vascular endothelial cells and autophagy. Autophagy is activated by DENV infection to prevent the fusion of autophagosomes with lysosomes. DENV replicates, assembles, and matures in autophagosomes, thus evading neutralizing antibodies during transport [10, 11].

Autophagy is a lysosomal-dependent degradation pathway in eukaryotic cells that regulates intracellular homeostasis and affects innate immune mechanisms by modulating pattern-recognition receptors and signal transduction associated with injury-related molecular patterns. Thus, intracellular pathogens (e.g., viruses and bacteria) may be specifically recognized and quickly targeted to the autophagy degradation pathway [12, 13]. Recent studies have shown that DENV-2-induced autophagy exhibits a protective effect on infected cells. Denv-2 activates the autophagy pathway to increase the replication of its RNA, whereas the inhibition of autophagy results in a significant decrease in viral replication [14, 15]. DENV can induce vascular leakage through macrophage migration inhibitor factor secretion and autophagy formation. Infectious autophagy-associated DF vesicles released by DENV-infected cells can protect viral RNA in vesicles and avoid antibody neutralization to promote viral transmission [11]. Previous work indicated that DENV-2 can induce primary human umbilical vein endothelial cells (HUVECs) to induce autophagy through the adenosine monophosphate-activated protein kinase (AMPK)/extracellular signal-regulated kinase (ERK)/mammalian target of rapamycin (mTOR) signaling pathway [16]. However, the mechanism through which DENV-2 activates tuberous sclerosis complex 2 (TSC2) via the AMPK and ERK1/2 pathways causing mTOR inhibition is unclear.

Tripartite motif-containing 22 (TRIM22), also known as Staf50, is an interferon-stimulated gene. Previous studies showed that the TRIM protein family is involved in a wide range of cellular processes, including apoptosis, cell cycle progression, and autophagy [17]. Autophagy is regulated by TRIM22 and has both antiviral and viral replication effects. On the one hand, TRIM22 promotes GEM-induced prosurvival autophagy and protects non-small cell lung cancer (NSCLC) cells from apoptosis [18]. TRIM22 promotes viral replication by regulating autophagy. Related studies have demonstrated that TRIM22 binds with the autophagy-related proteins, unc-51-like autophagy activating kinase 1 (ULK1), and Beclin1 to induce autophagy, thus promoting the replication of the respiratory syncytial virus (RSV) [19]. The preliminary proteomics results of the current study demonstrated that TRIM22 protein expression significantly increased 36 h after HUVECs were infected with DENV-2. A protein–protein interaction analysis in the Search Tool for the Retrieval of Interacting Genes/Proteins (STRING) database revealed that TRIM22 was associated with the AMPK/ERK/mTOR signaling pathway. Therefore, whether TRIM22 is involved in autophagy activation of DENV-2-infected HUVECs through the AMPK/ERK/mTOR pathway remains to be determined. Based on these findings, the mechanism of autophagy induced by the activation of the AMPK/ERK/mTOR signaling pathway following DENV-2 infection was explored. Determining how DENV-2 induces HUVEC autophagy through relevant signal transduction pathways will contribute to the development of effective immunotherapies and appropriate chemical drugs. In addition, this will provide an important theoretical basis for better understanding the pathogenic mechanism(s) of viral infection and lead to the development of antiviral drugs that target autophagy.

## Materials and methods

### Cell strain

The Denv-2 standard strain (NGC strain) was preserved in liquid nitrogen. HUVECs were purchased from ScienCell (Carlsbad, CA, USA). Aedes albopictus cells (C6/36) were purchased from the Kunming Cell Bank of the Chinese Academy of Sciences (Kunming, China). TOP10 competent *Escherichia coli* cells were purchased from the TIANGEN Company (Beijing, China).

### Plasmid

Br-v108 vectors (AgeI and EcoRI restriction enzyme cutting sites) were purchased from Shanghai Jikai Gene Technology Co., Ltd. (Shanghai, China). Lv-007 vectors (NheI and AgeI restriction enzyme cutting sites) were purchased from YBR BioSCI Res (Shanghai, China).

### Reagents and instruments

Extracellular matrix (ECM) media were purchased from ScienCell. RPMI-1640 media was purchased from Gibco (Thermo Fisher Scientific, Waltham, MA, USA). Trizol was purchased from Sigma-Aldrich (Sigma-Aldrich, Burlington, MA, USA). Hiscript QRT Supermix for quantitative PCR (qPCR) and AceQ qPCR SYBR Green was purchased from Vazyme (Nanjing, China). Antibodies (TRIM22, Beclin1, P62, ATG7, ATG5, mTOR, P-mTOR, AMPK, P-AMPK, ERK, P-ERK, and GAPDH) were purchased from Abcam (Abcam, Cambridge, UK). Age I and EcoRI were purchased from NEB (New England Biolabs, Ipswich, MA, USA). The BCA Protein Assay Kit was purchased from HyClone-Pierce, Inc (Thermo Fisher Scientific). Real-time fluorescence quantitative polymerase chain reaction (PCR) was purchased from ABI (StepOne PLUS, Thermo Fisher Scientific). The gel imager was purchased from Bio-Rad (Bio-Rad, Hercules, CA, USA). The ultraviolet–visible spectrophotometer was purchased from Thermo Fisher Scientific. The chemiluminescence imaging system used was a GE AI600. The microplate reader was purchased from Bio-Rad.

### Cell culture and identification

HUVECs stored in liquid nitrogen were removed and thawed in a 37 °C water bath. HUVECs were transferred to a T25 cm^2^ flask containing 5 mL of ECM complete culture solution (10% FBS, 1% ECGs, and 1% P/S) at 37 °C with 5% CO_2_. Cell growth was observed each day after HUVECs had adhered to the wall. The growth density of the HUVECs was 80–90% for subculture. The subculture conditions were 28 °C with 5% CO_2_ and ECM complete culture solution was the culture medium. When specific molecules, vWF/Factor VIII and CD31, were identified as positive by immunofluorescence, and HIV-1, HBV, HCV, mycoplasma, bacteria, yeast, and fungi were identified as negative, the first generation was cryopreserved when the number of cells was greater than 5 × 10^5^/mL. In this laboratory, subculture was continued to the fourth generation for the experiments.

### DENV-2 virulence test

*Aedes albopictus* cells (C6/36) were recovered, cultured, and subcultured. At the logarithmic growth stage (density, 80–90%), the cells were seeded into 96-well plates and cultured at 28 °C in a 5% CO_2_ atmosphere overnight. HUVECs stored in liquid nitrogen were removed and quickly thawed in a 37 °C water bath. The virus stock was diluted tenfold with an RPMI-1640 maintenance solution. Eight concentrations (10^−3^–10^−10^) and eight wells for each concentration were noted. The blank control was established and different dilutions of disease venom were simultaneously added and incubated at 37 °C with 5% CO_2_ for 2 h. The supernatant was discarded, the cells were washed with Hank’s solution, and a fresh medium (200 μL/well) was added. The culture was continued and cytopathic conditions were observed and recorded. The number of lesion holes at each dilution level was recorded and counted within 5 days and the Reed–Muench method was used to calculate the toxicity of DENV-2 to C6/36.

### DENV-2 infects HUVECs

HUVECs were recovered, cultured, and subcultured. The cells were seeded into six-well plates and cultured at 37 °C with 5% CO_2_ overnight at the logarithmic growth stage (density, 80–90%). DENV-2 was stored in liquid nitrogen, removed, and quickly thawed in a 37 °C water bath. The original virus solution was diluted 10^3^, 10^4^, and 10^5^ times with maintenance solution, and the blank control was established. The diluted disease venom was added and incubated at 37 °C with 5% CO_2_ for 2 h. The flask was shaken every 30 min to evenly distribute the venom. The supernatant was discarded and the cells were washed twice with Hank’s wash solution. A maintenance solution was added and the culture was continued for 36 h. Total protein from cells in the experimental and blank groups was collected.

### TEM detection of HUVEC autophagosomes

HUVECs were infected with DENV-2 virus stock solution, which was diluted 10^5^ times. Simultaneously, the blank control was set at 37 °C with 5% CO_2_ and the cells were collected at 24 and 36 h. The cells were analyzed by electron microscopy at the Chongqing Medical University after fixing with 2.5% glutaraldehyde. The ultrastructure of the cells was observed by TEM and imaged after dehydration, resin soaking, embedding, sectioning, and staining.

### *TRIM22* gene RNA interference interferes with plasmid vector construction

Using the *TRIM22* gene as a template, three RNA interference target sequences were designed and an RNA interference lentiviral vector was constructed. shTRIM22-1, shTRIM22-2, and shTRIM22-3 represent the knockdown shRNA targets at three different sites designed for the *TRIM22* gene. shRNA interference sequences were designed following the selected target sequences, and appropriate restriction enzyme sites were included at both ends. A double-stranded DNA oligo was prepared and linearized by Age I and EcoR I double digestion of the BR-V108 vector, ligated into the linearized vector, and transformed into TOP10-competent *E. coli* cells by heat shock. After screening, monoclonal expansion culture was conducted, the bacterial fluid was collected, and the plasmid was isolated using the Zyokang GTC endotoxin-free plasmid extraction kit. The plasmid concentration was measured using a microspectrophotometer. The plasmid was identified by 1% agarose gel electrophoresis after PCR amplification.

### Construction of a *TRIM22* gene-overexpressing lentiviral vector

The lV-007 vector was linearized by Nhe I and Age I double digestion. The target gene fragment was amplified by PCR. The homologous recombination sequence was added to the 5′ end of the amplimer, and the 5′- and 3′-terminal sequences of the amplified product matched that of the linearized clone vector. The linearized vector and the target gene fragment were recombined in vitro. The recombinant plasmid was transformed into the recipient cell, and individual clones were selected for identification by PCR and DNA sequencing. The positive clones were expanded and extracted to obtain plasmid with high purity.

### Lentivirus infection of HUVECs

HUVECs in the logarithmic phase (cell density reaching 80–90%) were divided into an shCtrl group (lentivirus empty vector transfection group, as negative control, NC control group) and an shTRIM22 group (TRIM22 knockdown group). The cells (1 × 10^5^) were inoculated cells into six-well plates and supplemented with EMC complete culture medium to 2 mL. The plates were mixed and incubated at 37 °C with 5% CO_2_ for 24 h. The supernatants were discarded and 1 mL of opti-MEM serum-free medium was added to each well. NC control virus and TRIM22 knockdown virus were added at 10^8^ TCID_50_/mL, shaken, and incubated at 37 °C with 5% CO_2_ for 18 h. The supernatant was discarded and 2 mL of ECM complete culture medium was added to each well for 48 h. The fluorescence intensity and infection efficiency were assessed by fluorescence microscopy.

### Quantitative real-time PCR detection

Total RNA was extracted using Trizol from the NC control virus infected with DENV-2 and HUVECs treated with TRIM22 knockdown virus during the logarithmic growth period. The concentration and purity of RNA were measured using an ultramicro-ultraviolet-visible spectrophotometer, and cDNA was obtained by reverse transcription using the RNA as a template. The expression of the target genes was measured by SYBR Green-based qPCR. The procedure was as follows: predenaturation at 95 °C for 1 min, denaturation at 95 °C for 10 s, annealing at 60 °C for 30 s, and repetition of this program for 40 cycles. A melt curve was also established for each gene. Relative quantitative analysis *F* = 2^−△△Ct^ was used, where △Ct represents the Ct value of the target gene minus the Ct value of the reference gene, − △△Ct is the average value of △Ct in the group C–△Ct value of each sample. In addition, 2^−△△Ct^ reflects the relative expression level of target genes in each sample compared with the NC group. GAPDH expression was used as an internal reference. The primiers used refer to Table 1.

**Table 1:** Sequence of correlated primers for experiments

Gene	Primer	Length (bp)
P62	F: GCAATGGGCCTGTGGTAG	191
	R: CCCGAAGTGTCCGTGTTT	
ATG7	F: GTTGTTTGCTTCCGTGAC	142
	R: TGCCTCCTTTCTGGTTCT	
ATG5	F: AAGCAACTCTGGATGGGATT	173
	R: GCAGCCACAGGACGAAAC	
TRIM22	F: GAGATGTCTGTGAGCACCAT	136
	R: TCCTTGACCACCTCGTTT	
Beclin1	F: CGTGGAATGGAATGAGAT	110
	R: CGTAAGGAACAAGTCGGTAT	
ERK	F: TGTTCCCAAATGCTGACT	131
	R: GGGTCGTAATACTGCTCC	
AMPK	F: CCGAGAAGCAGAAACACG	167
	R: CACATCAAGGCTCCGAAT	
mTOR	F: GCTGTCATCCCTTTATCG	100
	R: TCTTCTTCTTCTCCCTGTAGTC	
GAPDH	F: TGACTTCAACAGCGACACCCA	121
	R: CACCCTGTTGCTGTAGCCAAA	

### Cell cycle assay

HUVECs in the logarithmic growth stage were counted and the cells were seeded into six-well plates and cultured with NC control virus and TRIM22 knockdown virus at 10^8^ TCID_50_/mL. After the cells of the HUVEC NC and TRIM22 knockdown groups reached 90%, 300 μL of ECM medium was added to each well to prepare a cell suspension. The cells were collected in 5 mL centrifuge tubes with three wells per group. After centrifugation at 300 g for 5 min, the supernatant was discarded and the propidium iodide staining solution was added to resuspend the cells. The cells were analyzed by flow cytometry (FCM) after incubating in the dark for 20 min.

### CCK8 assay for cell proliferation

HUVECs were seeded into six-well plates and cultured with NC control and TRIM22 knockdown viruses at 10^8^ TCID_50_/mL. After cell fusion, the HUVEC NC and TRIM22 knockdown groups were cultured to a density of 90% and counted. The NC and TRIM22 knockdown groups were then inoculated into 96-well plates at 5 × 10^3^ cells/100 μL with three wells for each group. Next, 10 μL of CCK8 solution was added to each well, mixed, and incubated at 37 °C with 5% CO_2_ for 4 h. The absorbance at 450 nm was measured with a microplate reader and the experimental data were recorded.

### Cell apoptosis

HUVECs were seeded into six-well plates and cultured with NC control and TRIM22 knockdown viruses at 10^8^ TCID_50_/mL. After the cells reached 90% confluence, the cell suspensions were collected, centrifuged at 300 g for 3 min, and resuspended with 1 mL of 1 × binding buffer. Then, 5 μL of Annexin V-APC dye was added to each tube and mixed gently, followed by the addition of 10 μL of PI dye. The cells were analyzed within 1 h by FCM after incubating for 20 min on ice at room temperature without light.

### Western blot analysis

Cells in the logarithmic phase were collected from each group and washed twice with Hank’s solution. The residual solution was discarded, and the cells were lysed in RIPA buffer (PMSF:RIPA = 1:100) containing protease and phosphoprotease inhibitors. The cells were shaken and lysed on ice, scraped off at 4 °C, and centrifuged at 12,000 rpm for 30 min. The supernatant was transferred to 1.5 mL centrifuge tubes and the concentration of each protein sample was measured by the BCA method. A 5× protein loading buffer was added to 40 μg of total protein for each sample. The proteins were separated by sodium dodecyl sulfate–polyacrylamide gel electrophoresis at 80 V for 20 min followed by 120 V for 100 min. Following the transfer, the PVDF membranes were blocked with 5% skim milk for 120 min and incubated with primary antibody overnight. TBST was used to wash the membranes six times for 5 min each. The secondary antibody was added and incubated at room temperature for 120 min on a shaking table. After chemiluminescent detection, Image J software was used to analyze the relative amount of each protein band. The additional file 1 provided is the original data of Western Blot, and these results have been show in Figs. 8, 9, 13, 14, 17, 18 respectively.

### Statistical analysis

The experimental data were analyzed by Statistical Product and Service Solutions 25.0 (SPSS25.0). All experiments were repeated at least three times. The data were plotted using GraphPad Prism 8.0 software. The data that conformed to a normal distribution were expressed as the mean ± standard deviation $\left( {\overline{{\text{X}}} \pm {\text{s}}} \right)$. One-way analysis of variance was used for comparisons between groups, and a Student’s *t*-test was used for pairwise comparison. *P* < 0.05 was considered statistically significant.

## Results

### TRIM22 protein expression increases in HUVECs after DENV-2 infection

**Fig. 1:**
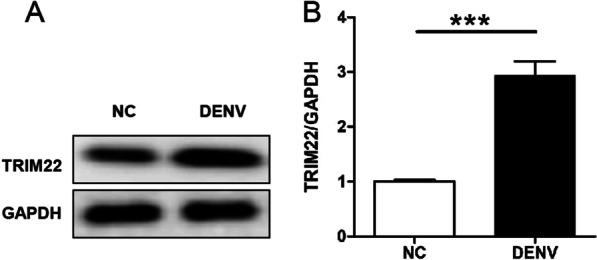
**A** The protein expression of TRIM22 in DENV-2-infected HUVECs was detected in western blot analysis; **B** The quantitative analysis of TRIM22 expression. ***P < 0.001

DENV-2 at a concentration of 10^5^ TCID_50_/mL was used to infect HUVECs for 36 h. The expression of HUVEC TRIM22 protein was determined by western blot analysis. Compared with the control group, TRIM22 protein expression in the DENV-2-infected group significantly increased (*P* < 0.001) (Fig. 1A, B).

### qRT-PCR analysis of interference targets of TRIM22

**Fig. 2:**
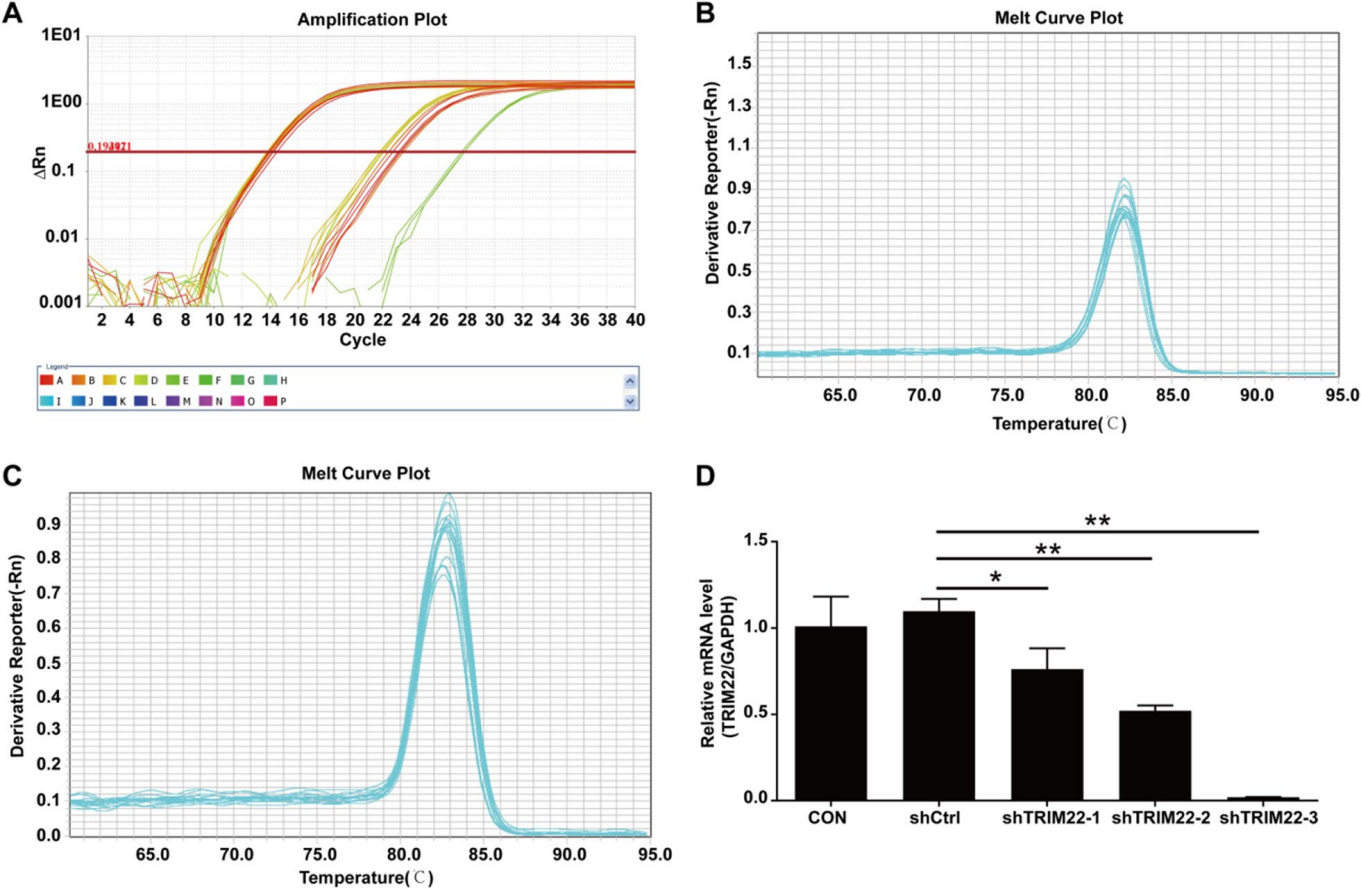
Screening of effective interference targets by qRT-PCR. **A** Amplication plot, Letters A–P represent different samples; **B** Melt curve plot of H-GAPDH; **C** Melt curve plot of H-TRIM22; **D** The knockdown efficiency of sh-TRIM22-1, sh-TRIM22-2 and sh-TRIM22-3 was detected by RT-qPCR. shTRIM22-1, shTRIM22-2, and shTRIM22-3 were used to target TRIM22. *P < 0.05, **P < 0.01

The qRT-PCR results indicated that compared with the shCtrl group, the *TRIM22* gene knockdown efficiency in the shTRIM22-1, SHTRIM22-2, and SHTRIM22-3 groups were 30.6% (*P* < 0.05), 52.5% (*P* < 0.01), and 98.1% (*P* < 0.01), respectively. Therefore, the shTRIM22-3 group was used to conduct subsequent experiments (Fig. 2A–D).

### Knockdown efficiency detection

**Fig. 3:**
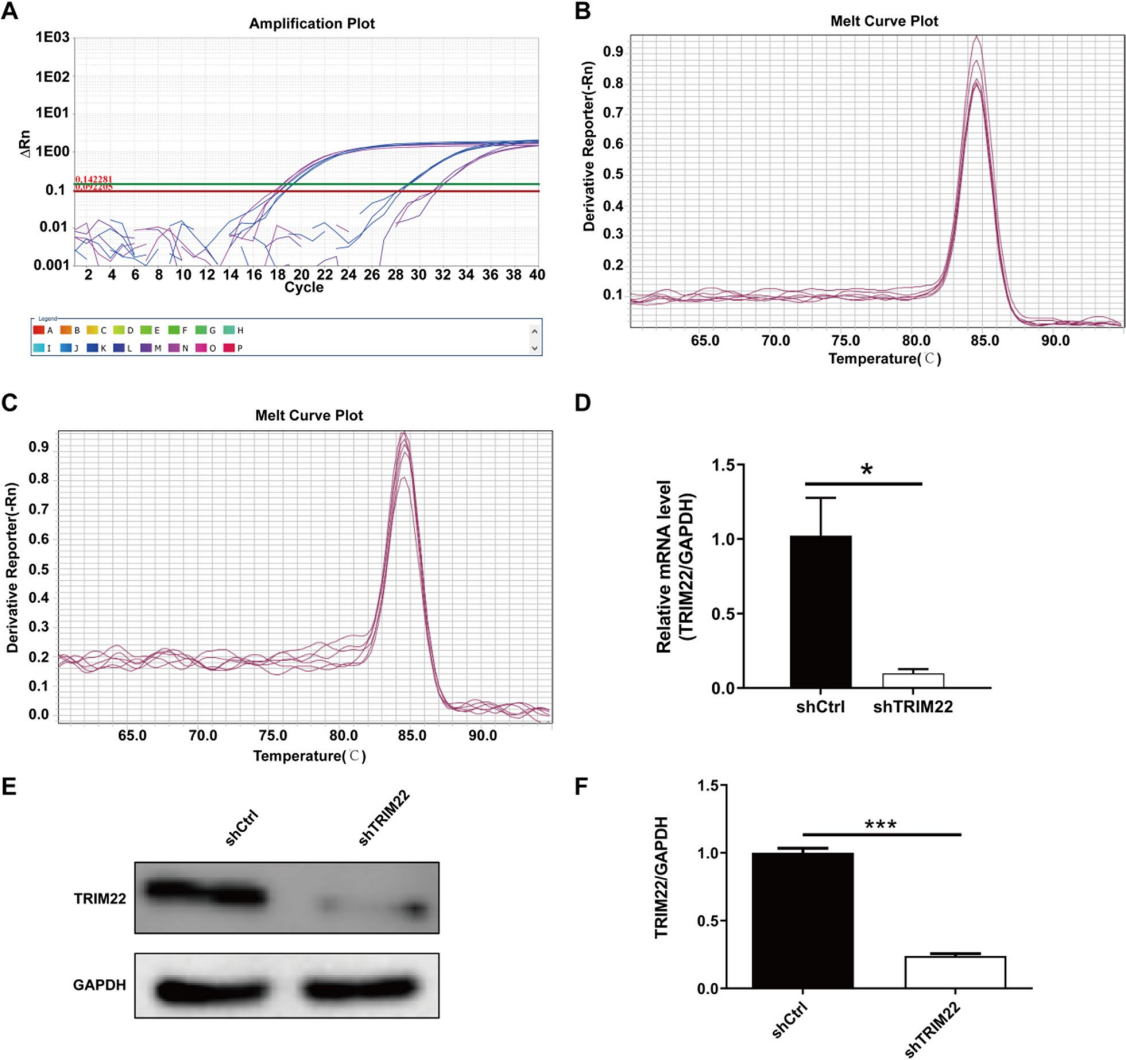
Knockdown efficiency detection. **A** Amplication plot, Letters A–P represent different samples; **B** Melt curve plot of H-GAPDH; **C** Melt curve plot of H-TRIM22; **D** The mRNA expression of TRIM22 after TRIM22 knockdown was detected by RT-qPCR; **E**, **F** The protein expression of TRIM22 after TRIM22 gene knockdown was detected by western blot analysis. *P < 0.05, ***P < 0.001

The qRT-PCR results indicated that, compared with the shCtrl group, the knockdown efficiency of shTRIM22 on HUVECs was 90.43% (*P* < 0.05) (Fig. 3A–D). Western blot analysis confirmed that, compared with the shCtrl group, the expression of TRIM22 protein in the shTRIM22 group significantly decreased (*P* < 0.001), indicating that TRIM22 knockdown was successful (Fig. 3E–F).

### Lentivirus infects HUVECs

**Fig. 4:**
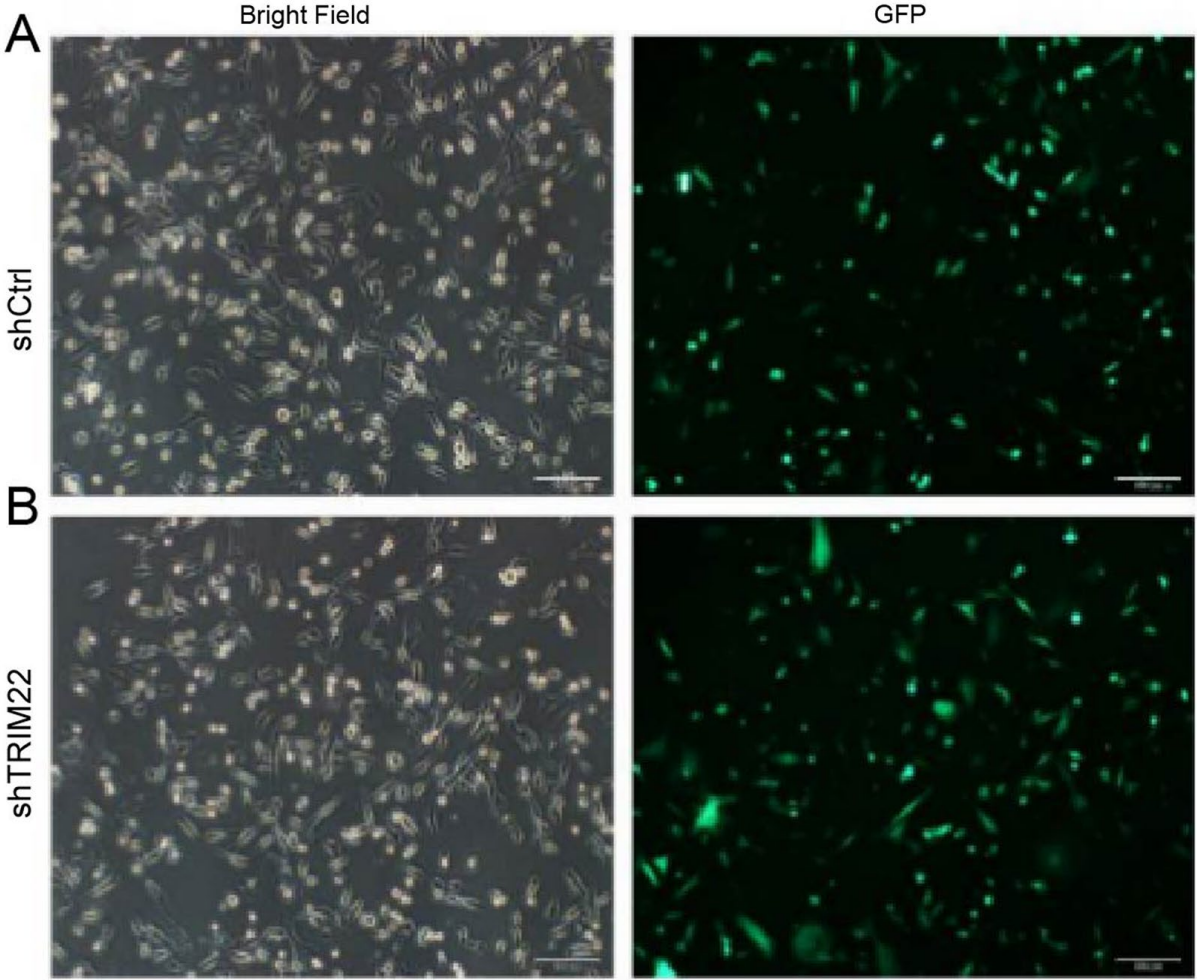
Lentivirus infection of HUVEC (200×). **A**, **B** The GFP fluorescence was evaluated in shCtrl or shTRIM22-infected HUVECs

Significant green fluorescence was observed by microscopy after a 72-h infection of HUVECs with lentivirus in the shCtrl or shTRIM22 groups. The results indicated that the infection efficiency was > 80% and the cell morphology was normal (Fig. 4A, B).

### Effect of shTRIM22 on cell proliferation

**Fig. 5:**
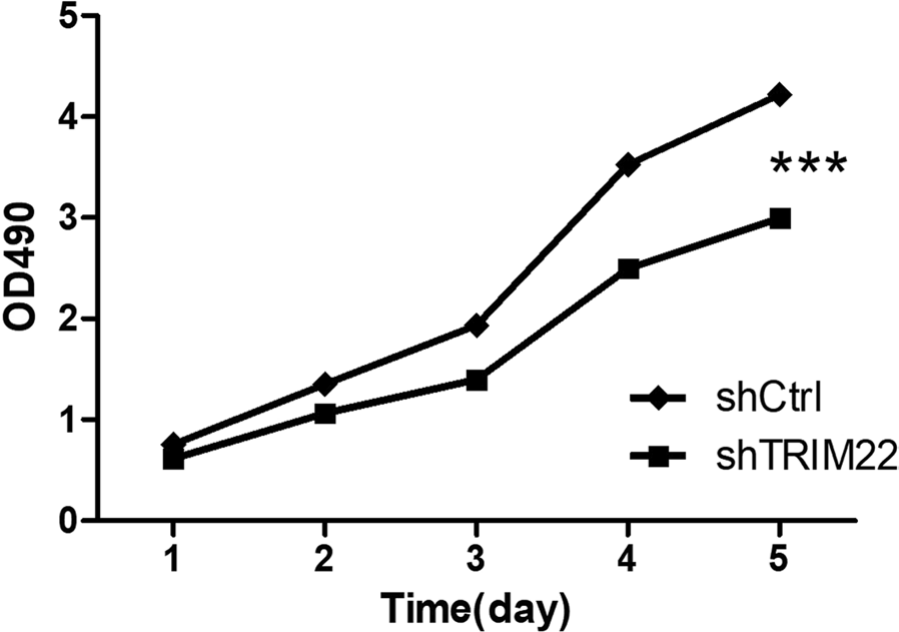
The effect of shTRIM22 on cell proliferation as detected by CCK-8 assay. ***P < 0.001

The *TRIM22* gene was used to construct a TRIM22 knockdown lentivirus vector, which was transfected into HUVECs. The CCK8 method was used to assess changes in cell viability over five consecutive days. The cell proliferation results are shown in Fig. 5. After lentivirus infection, compared with the shCtrl group, the OD value of the shTRIM22 group was smaller at the same time point, indicating that the proliferation rate of the HUVECs in the shTRIM22 group was slower (Fig. 5). The difference was statistically significant (*P* < 0.001), indicating that TRIM22 affects HUVEC proliferation.

### Ultrastructure of HUVEC autophagosomes after TRIM22 knockdown using TEM

**Fig. 6:**
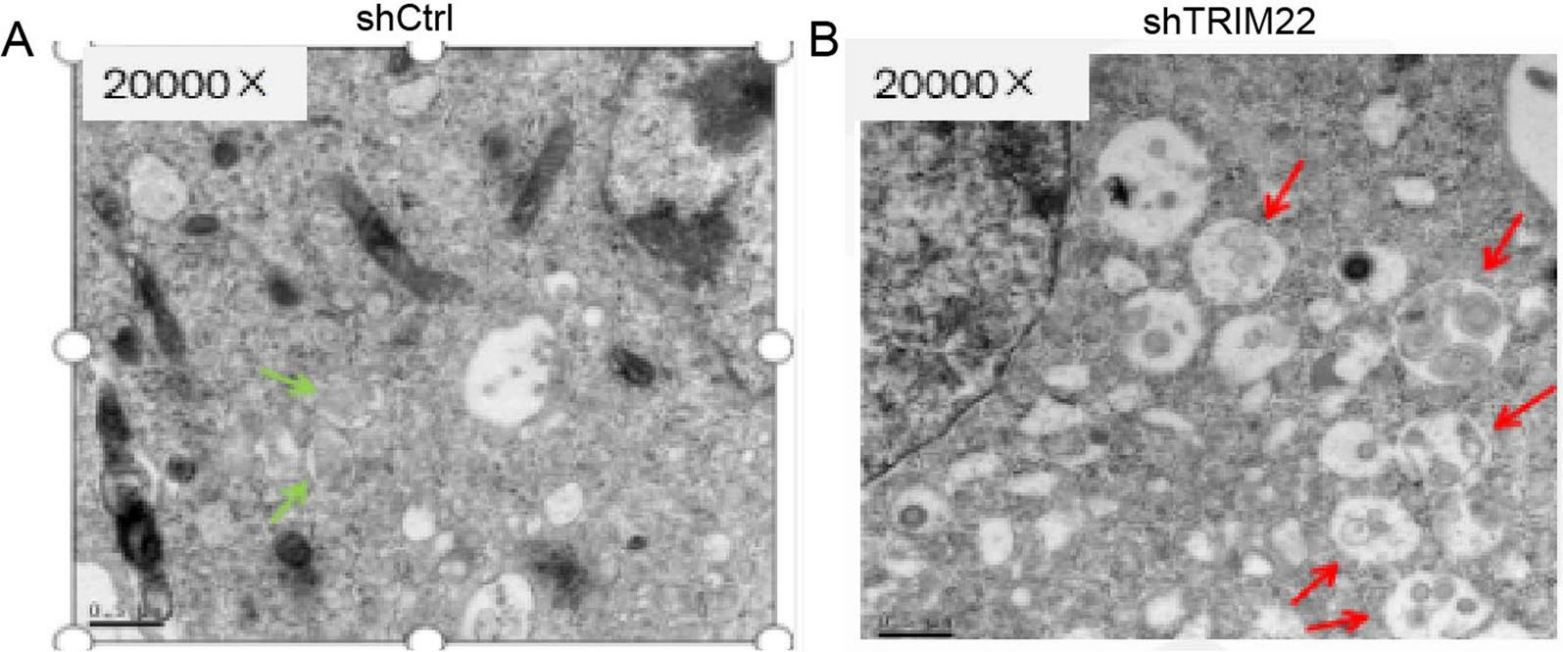
**A**, **B** The ultrastructure of HUVEC autophagy observed by TEM in shCtrl and shTRIM22 groups. Green arrow: autophagosome, red arrow: autolysosome

Figure 6 shows scattered vacuoles observed in both the shCtrl and shTRIM22 groups, which were surrounded by a membrane. The nuclear fragments and organelles were evident in the small body. However, compared with the shCtrl group, the ultrastructure of HUVEC autophagosomes following TRIM22 knockdown showed increased autophagolysosomes and reduced autophagy, indicating that TRIM22 knockdown significantly affects autophagy in HUVECs (Fig. 6A, B).

### TRIM22 knockdown affects the expression of HUVEC autophagy and AMPK pathway-related genes

**Fig. 7:**
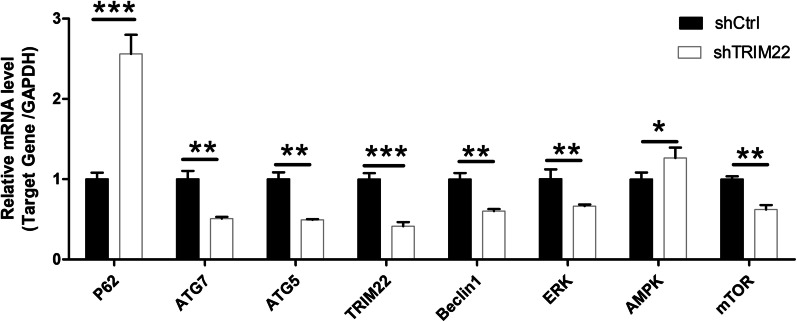
Expression of HUVEC autophagy and AMPK pathway-related genes after TRIM22 knockdown. *P < 0.05, **P < 0.01, ***P < 0.001

The qRT-PCR results indicated that, compared with the shCtrl group, the expression of ATG7, ATG5, Beclin1, ERK, and mTOR genes in HUVECs with TRIM22 knockdown (*P* < 0.01) as well as the expression of *TRIM22* genes (*P* < 0.001) were downregulated. In contrast, the mRNA expression of AMPK (*P* < 0.05) and *P62* (*P* < 0.001) genes was upregulated (Fig. 7). These results indicate that TRIM22 upregulates the expression of autophagy and AMPK signaling pathway-related genes in HUVECs.

### TRIM22 knockdown affects the expression of autophagy-related proteins in HUVECs

**Fig. 8:**
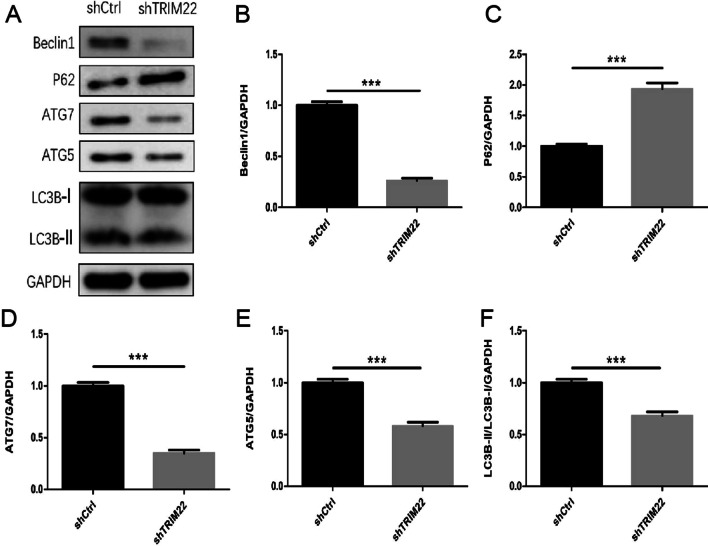
Expression of autophagy-related proteins. **A** The effect of TRIM22 knockdown on the expressions of autophagy-related proteins in HUVECs as detected by western blot analysis; **B**–**F** The quantitative expressions analysis of autophagy-related proteins. **P < 0.01, ***P < 0.001

Compared with the shCtrl group, the expression of ATG5, ATG7, Beclin1, and LC3B-II protein in HUVECs with TRIM22 knockdown decreased (*P* < 0.001), whereas P62 protein expression was increased (*P* < 0.01) (Fig. 8A–F). This indicates that autophagy was inhibited following TRIM22 knockdown and shows that TRIM22 induces autophagy.

### TRIM22 knockdown affects the expression of AMPK pathway-related proteins

**Fig. 9:**
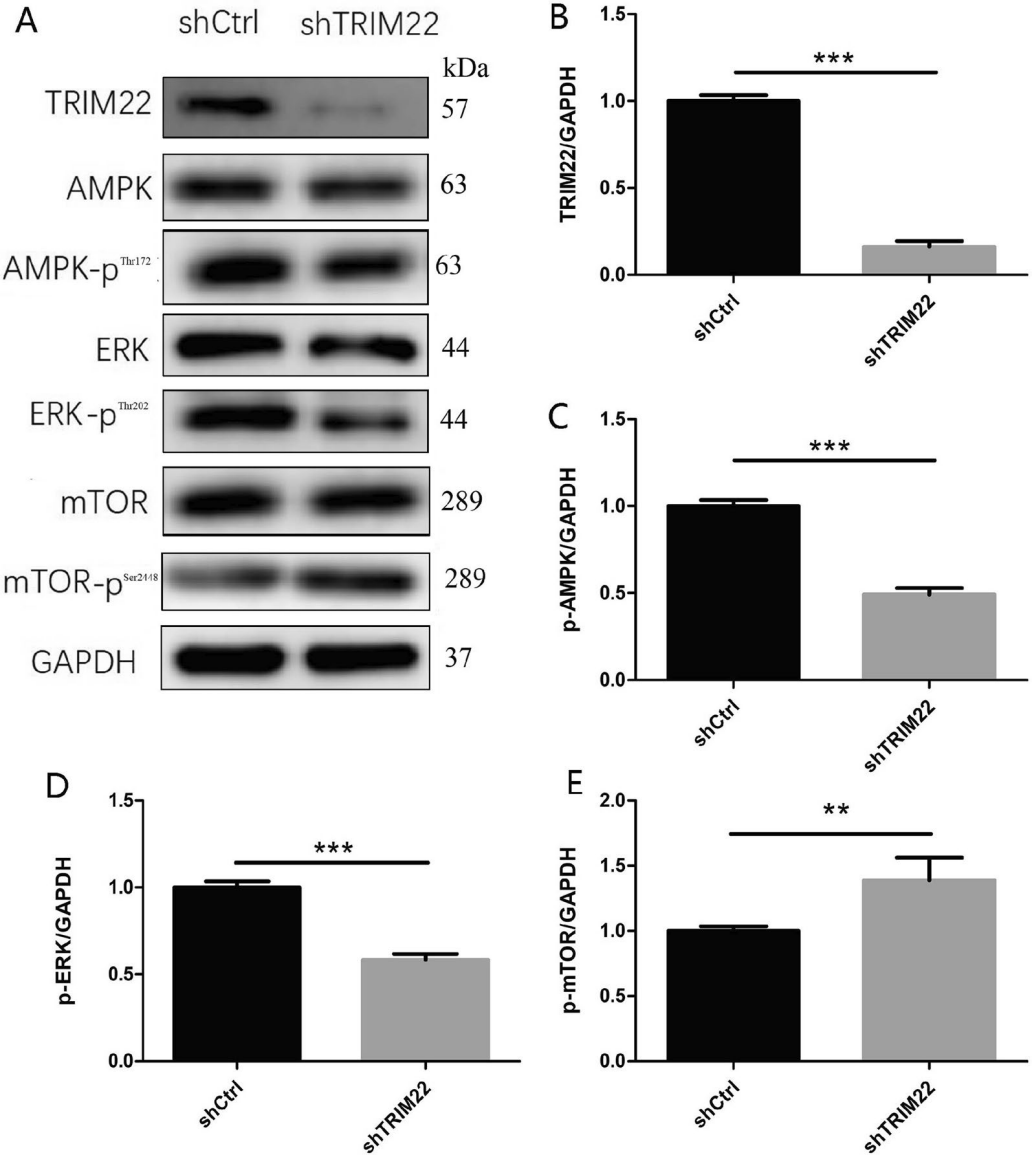
Expression of AMPK pathway-related proteins. **A** The effect of TRIM22 knockdown on the expressions of AMPK pathway-related proteins in HUVECs as detected by western blot analysis; **B**–**E** The quantitative expressions analysis of AMPK pathway-related proteins. **P < 0.01, ***P < 0.001

Compared with the shCtrl group, the expression of p-AMPK and p-ERK decreased in HUVECs with TRIM22 knockdown (*P* < 0.001), whereas p-mTOR expression increased (*P* < 0.01) (Fig. 9A–E), indicating that TRIM22 may have a positive regulatory effect on the AMPK/ERK/mTOR pathway.

### Effect of TRIM22 knockdown on the cell cycle

**Fig. 10:**
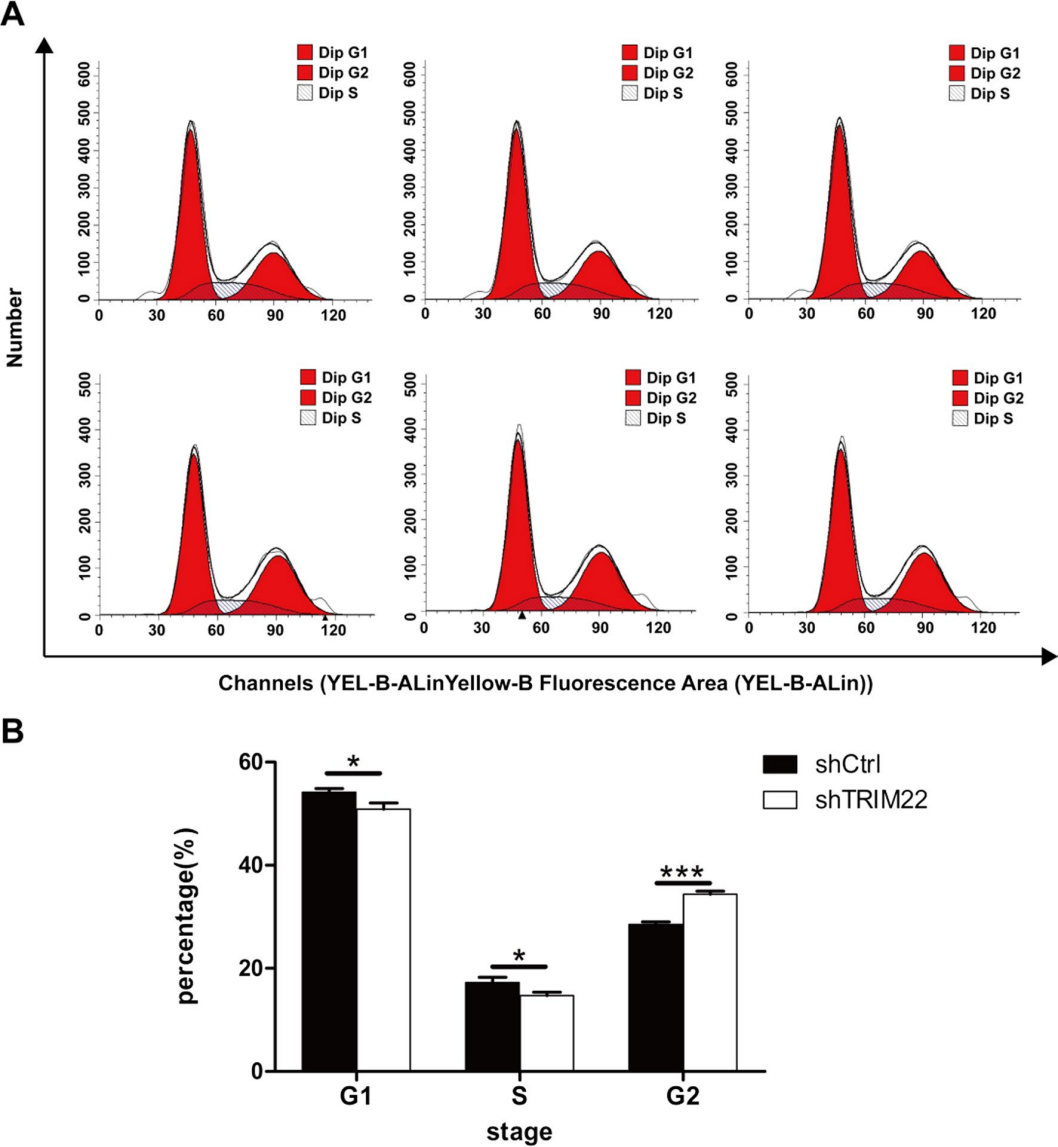
Detection of cell cycle. **A** The effect of TRIM22 knockdown on the cell cycle of DENV-2-infected HUVECs, the abscissa is the fluorescence intensity of PI, which represents the DNA content of the cells, and the ordinate is the number of cells; **B** the histogram analysis of each phase of the cell cycle. *P < 0.05, ***P < 0.001

Intracellular DNA content was stained with PI and subjected to FCM for cell cycle analysis. Figure 10 shows that the proportion of HUVECs in the G1/G0 phase decreased (*P* < 0.05) and that in the S phase decreased (*P* < 0.05) compared with the shCtrl group 36 h after DENV-2 treatment of HUVECs with TRIM22 knockdown. An increased cell number in the G2/M phase was also observed (28.53%/34.36%, *P* < 0.001). TRIM22 knockdown blocked the DNA synthesis phase (S phase) and promoted the late DNA synthesis phase (G2/M phase) (Fig. 10A, B).

### Effect of TRIM22 knockdown on apoptosis in HUVECs infected with DENV-2

**Fig. 11:**
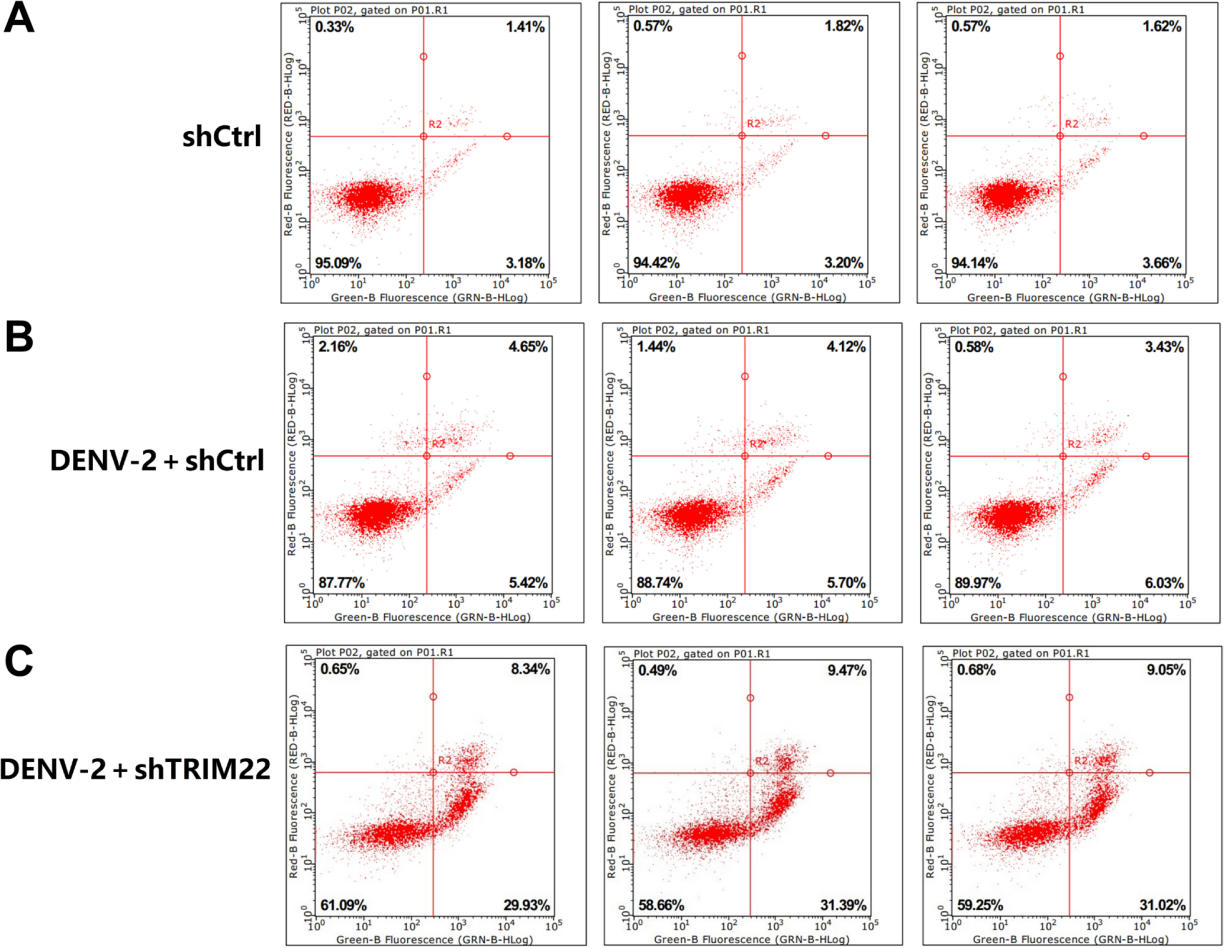
Effect of TRIM22 knockdown on HUVEC apoptosis following DENV-2 infection. ShCtrl, negative control group; DENV-2 + shCtrl, negative control group for DENV-2 infection; DENV-2 + vshTRIM22, DENV-2 infected HUVECs with TRIM22 knockdown

FCM was used to determine the effect of TRIM22 knockdown on apoptosis in HUVECs infected with DENV-2 (Fig. 11). The results indicated that the total apoptosis rate of the shCtrl group was 4.59% (early apoptosis, 3.18%; late apoptosis, 1.41%) and that of DENV-2 + shCtrl group was 10.07% (early apoptosis, 5.42%; late apoptosis, 4.65%). The total apoptosis rate of the DENV-2 + shTRIM22 group was 40.86% (early stage, 31.39%; late stage, 9.47%). This suggests that HUVECs with TRIM22 knockdown contain more early apoptotic cells (LR) and late apoptotic cells (UR) compared with the control group. The total apoptosis rate (UR + LR) of the HUVECs with TRIM22 knockdown was also significantly higher compared with the control group (*P* < 0.001) (Fig. 11A–C). These results indicated that TRIM22 knockdown promotes apoptosis of HUVECs infected with DENV-2.

### Autophagy ultrastructure of DENV-2-infected HUVECs with TRIM22 knockdown using TEM

**Fig. 12:**
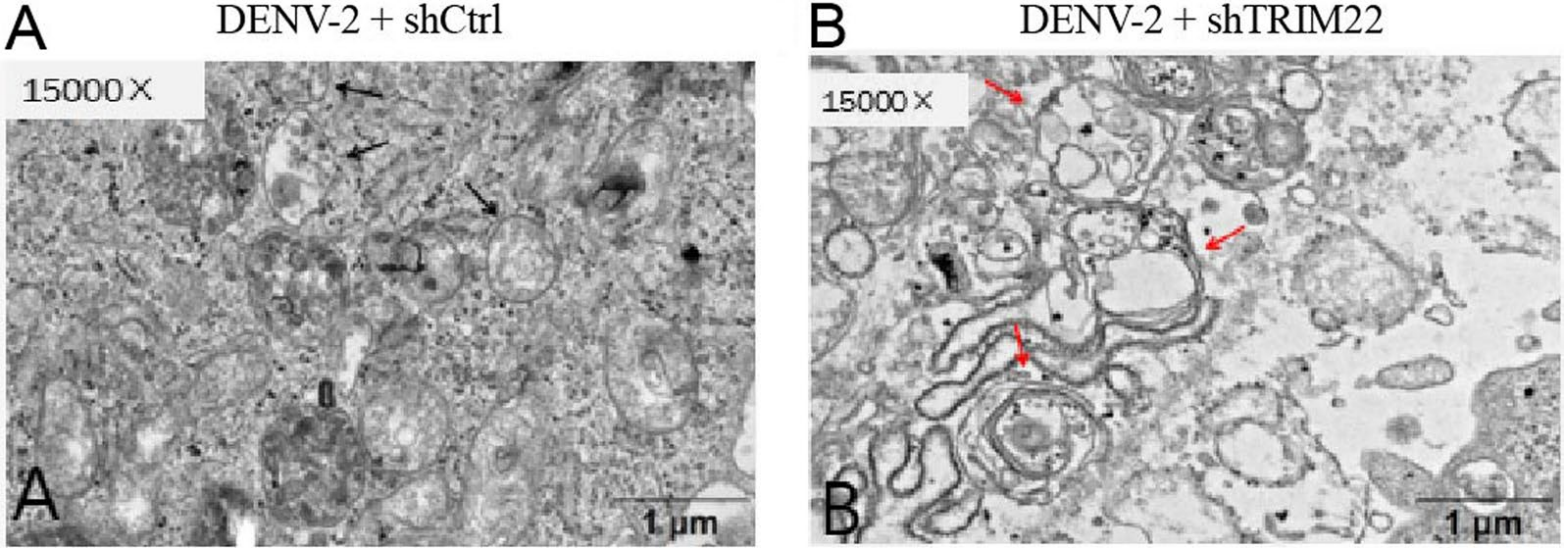
The ultrastructure of DENV-2-infected HUVECs after TRIM22 knockdown as observed by TEM. **A** Control group infected with DENV-2; **B** DENV-2-infected HUVEC group with TRIM22 knockdown. Black arrow autophagosome; red arrow autolysosome

TEM results showing the ultrastructural morphology of HUVECs are shown in Fig. 12. Scattered vacuoles surrounded by a membrane in each group of cells were noted, and nuclear fragments and organelles were observed in small bodies. Compared with the shCtrl group, HUVEC autolysosomes increased after TRIM22 knockdown (Fig. 12A, B). The results indicate that autophagy of HUVECs was inhibited following TRIM22 knockdown.

### Effects of TRIM22 knockdown on DENV-2-induced autophagy and AMPK pathway-related protein expression

**Fig. 13:**
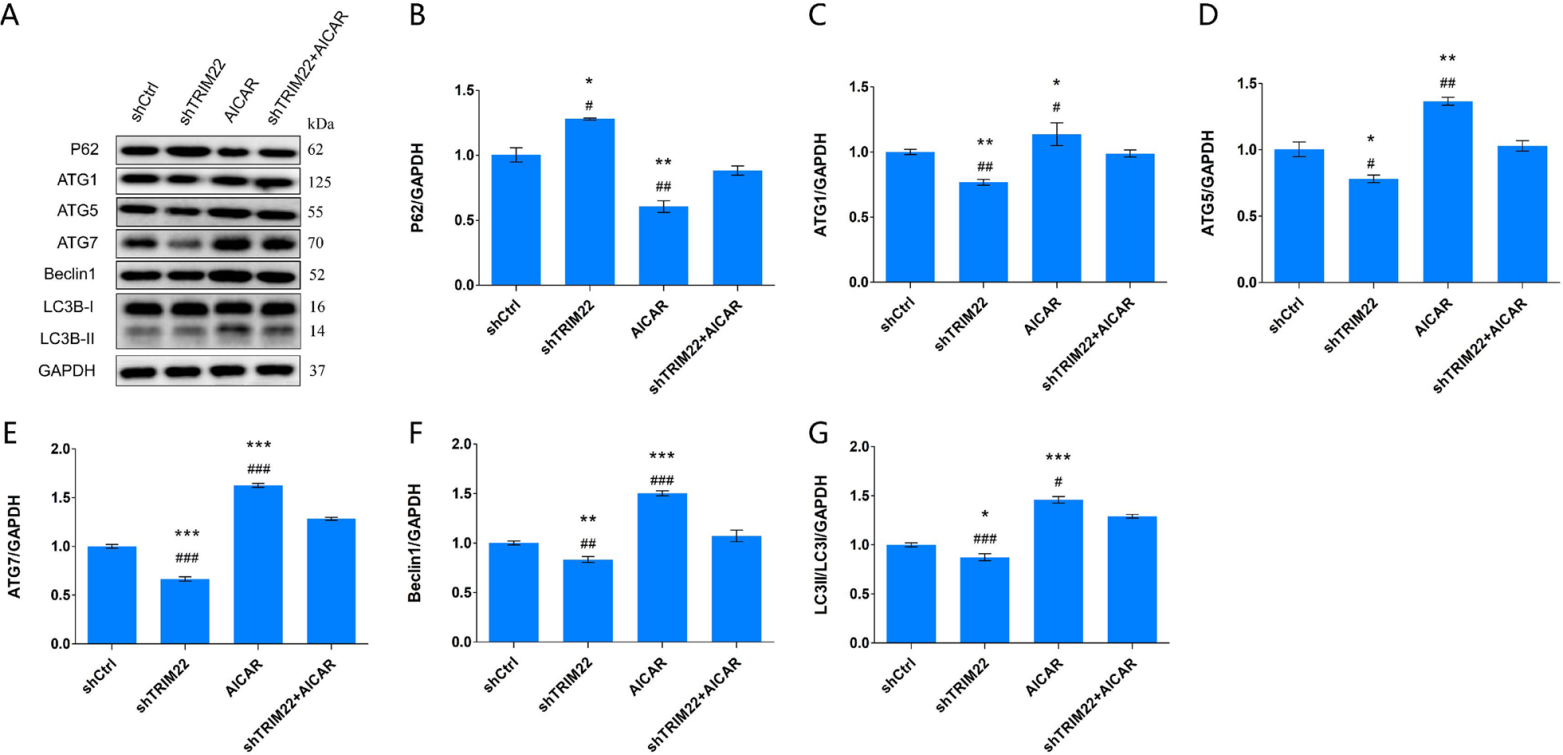
Effect of TRIM22 knockdown on autophagy-related factors. **A** The effect of TRIM22 knockdown on autophagy-related factors in DENV-2-infected HUVECs as detected by western blot analysis; **B**–**G** The quantitative expressions analysis autophagy-related proteins. shCtrl group: DENV-2 + shCtrl group; shTRIM22 group: DENV-2 + shTRIM22 group; AICAR group: DENV-2 + shCtrl + AICAR group; shTRIM22 + AICAR group: DENV-2 + TRIM22 + AICAR group. *represents comparison with the shCtrl group; *P < 0.05, **P < 0.01, and ***P < 0.001. #represents comparison with the shTRIM22 + AICAR group; #*P* < 0.05, ##*P* < 0.01, and ###*P* < 0.001

Whether the regulatory effect of TRIM22 on HUVEC autophagy occurs during DENV-2 infection was further explored. Thus, negative control (shCtrl), TRIM22 knockdown (shTRIM22), AMPK activator (AICAR), and TRIM22 knockdown + AMPK activator (shTRIM22 + AICAR) groups were established. All groups were infected with DENV-2 for 36 h, except for the negative control group. Western blot analysis was used to determine the effects of TRIM22 knockdown on the expression of autophagy proteins ATG1, ATG5, ATG7, Beclin1, and P62 and the activation of AMPK, ERK, and mTOR proteins of the AMPK/ERK/mTOR pathway in HUVECs. The results indicated that TRIM22 induced autophagy in HUVECs following DENV-2 infection. TRIM22 knockdown during DENV-2 infection reduced autophagy in HUVECs (Fig. 13A–G). These results suggest that TRIM22 promotes autophagy in HUVECs following DENV-2 infection, whereas TRIM22 knockdown reduces the induction of autophagy through the AMPK/ERK/mTOR signaling pathway.

### Effect of TRIM22 knockdown on DENV-2-induced HUVEC autophagy

Compared with the shCtrl group, the shTRIM22 group exhibited reduced expression of ATG1 (*P* < 0.01), ATG5 (*P* < 0.05), ATG7 (*P* < 0.001), and Beclin1 (*P* < 0.01) following DENV-2 infection of HUVECs with TRIM22 knockdown, whereas P62 expression levels increased (*P* < 0.05). These results suggest that TRIM22 knockdown has an inhibitory effect on DENV-2-induced autophagy. In the AICAR group, ATG1 (*P* < 0.05), ATG5 (*P* < 0.01), ATG7 (*P* < 0.001), and Beclin1 (*P* < 0.001) expression increased, whereas P62 expression decreased (*P* < 0.01). These results indicate that the expression of autophagy proteins following DENV-2-infection of HUVECs was enhanced following stimulation with an AMPK activator. In the shTRIM22 + AICAR group, compared with the shTRIM22 group, the expression levels of ATG1 (*P* < 0.05), ATG5 (*P* < 0.01), ATG7 (*P* < 0.001), Beclin1 (*P* < 0.001), and LC3II/LC3I (*P* < 0.05) increased, whereas P62 expression levels decreased (*P* < 0.01). Compared with the AICAR group, in the shTRIM22 + AICAR group, the expression levels of ATG1 (*P* < 0.01), ATG5 (*P* < 0.05), ATG7 (*P* < 0.001), Beclin1 (*P* < 0.001), and LC3II/LC3I (*P* < 0.001) decreased, whereas the P62 expression levels increased (*P* < 0.05). These results suggest that TRIM22 knockdown reduces autophagy induced by DENV-2 infection combined with an AMPK activator in HUVECs (Fig. 13A–G).

### Effect of TRIM22 knockdown on AMPK pathway-related proteins in DENV-2-infected HUVECs

**Fig. 14:**
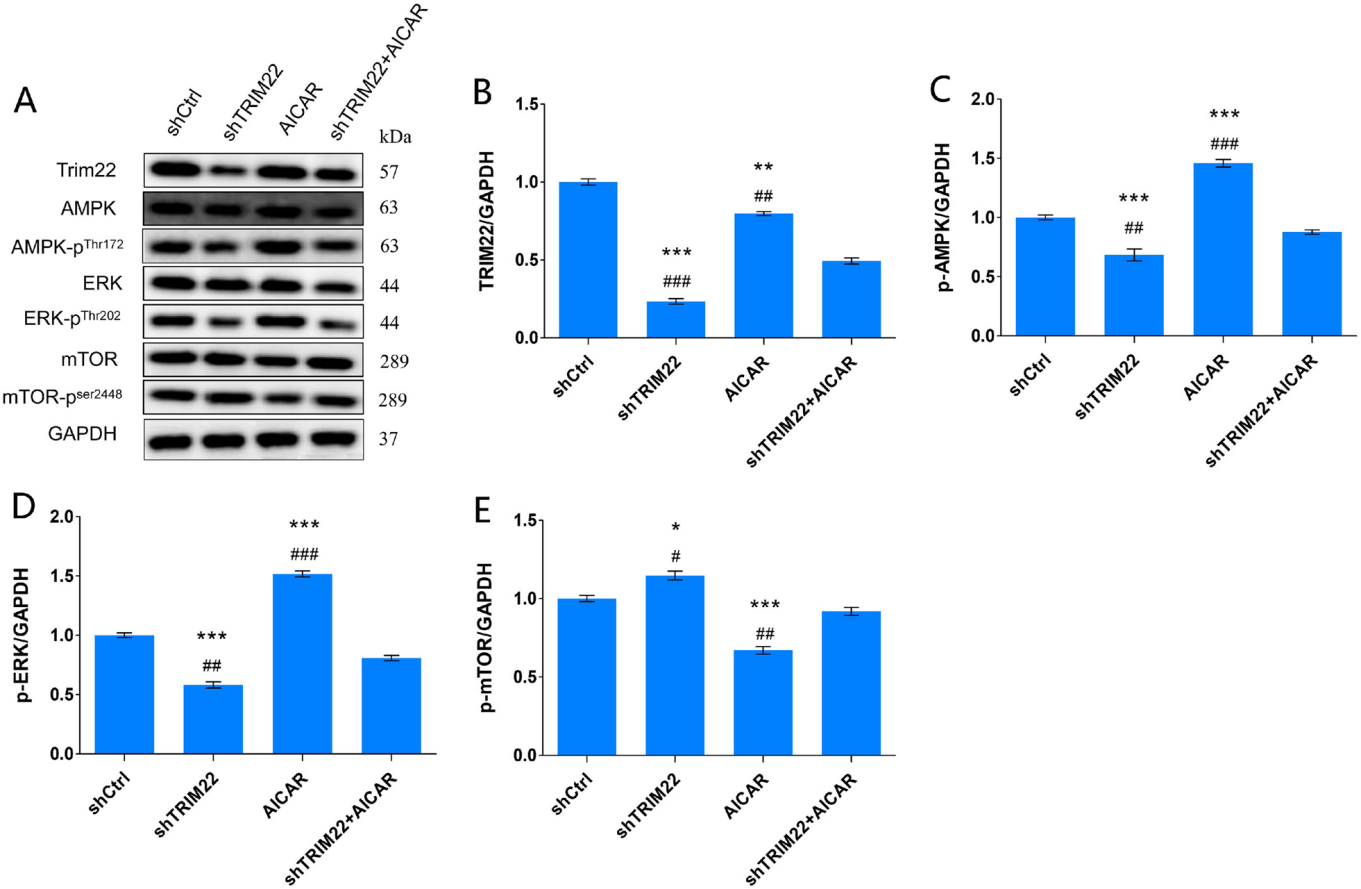
Effects of TRIM22 knockdown on expression of AMPK/ERK/mTOR pathway-related proteins. **A** The effect of TRIM22 knockdown on the expressions of AMPK/ERK/mTOR pathway proteins in DENV-2-infected HUVECs as detected by western blot analysis; **B**–**E** The quantitative expressions analysis of AMPK pathway-related proteins. *represents comparison with the shCtrl group; *P < 0.05, **P < 0.01, and ***P < 0.001. #represents comparison with the shTRIM22 + AICAR group; #*P* < 0.05, ##*P* < 0.01, and ###*P* < 0.001

Compared with the shCtrl group, following DENV-2 infection of HUVECs with TRIM22 knockdown, the expression of p-AMPK and p-ERK in the shTRIM22 group decreased (*P* < 0.01), whereas p-mTOR expression increased (*P* < 0.05). This indicates that TRIM22 knockdown downregulates the AMPK/ERK/mTOR signaling pathway. In the AICAR group, the expression of p-AMPK and p-ERK protein increased (*P* < 0.001), whereas p-mTOR protein expression decreased (*P* < 0.001). This suggests that AMPK/ERK/mTOR signaling is upregulated following stimulation with an AMPK activator. Compared with the AICAR group, the expression of p-AMPK and p-ERK in the shTRIM22 + AICAR group decreased (*P* < 0.01) and p-mTOR expression increased (*P* < 0.05). Compared with the shTRIM22 group, the expression of p-AMPK and p-ERK in the shTRIM22 + AICAR group increased (*P* < 0.001), whereas p-mTOR protein expression decreased (*P* < 0.01). These results suggest that TRIM22 knockdown inhibits the activity of the AMPK/ERK/mTOR pathway induced by DENV-2 infection combined with an AMPK activator (Fig. 14A–E).

### Effects of TRIM22 overexpression on DENV-2-infected HUVEC autophagy and AMPK pathway-related proteins

The Empty vector, TRIM22-OE, Dorsomorphin, and TRIM22-OE + Dorsomorphin groups were established to confirm the regulatory effect of TRIM22 on autophagy. All groups were infected with DENV-2 for 36 h, except for the shCtrl group. Western blot analysis was used to detect the HUVEC autophagy and the expression of AMPK pathway-related proteins. The results showed that TRIM22 promotes HUVEC autophagy induced by DENV-2 infection through positive regulation of the AMPK/ERK/mTOR signaling pathway.

### Establishment of a TRIM22 overexpression plasmid

**Fig. 15:**
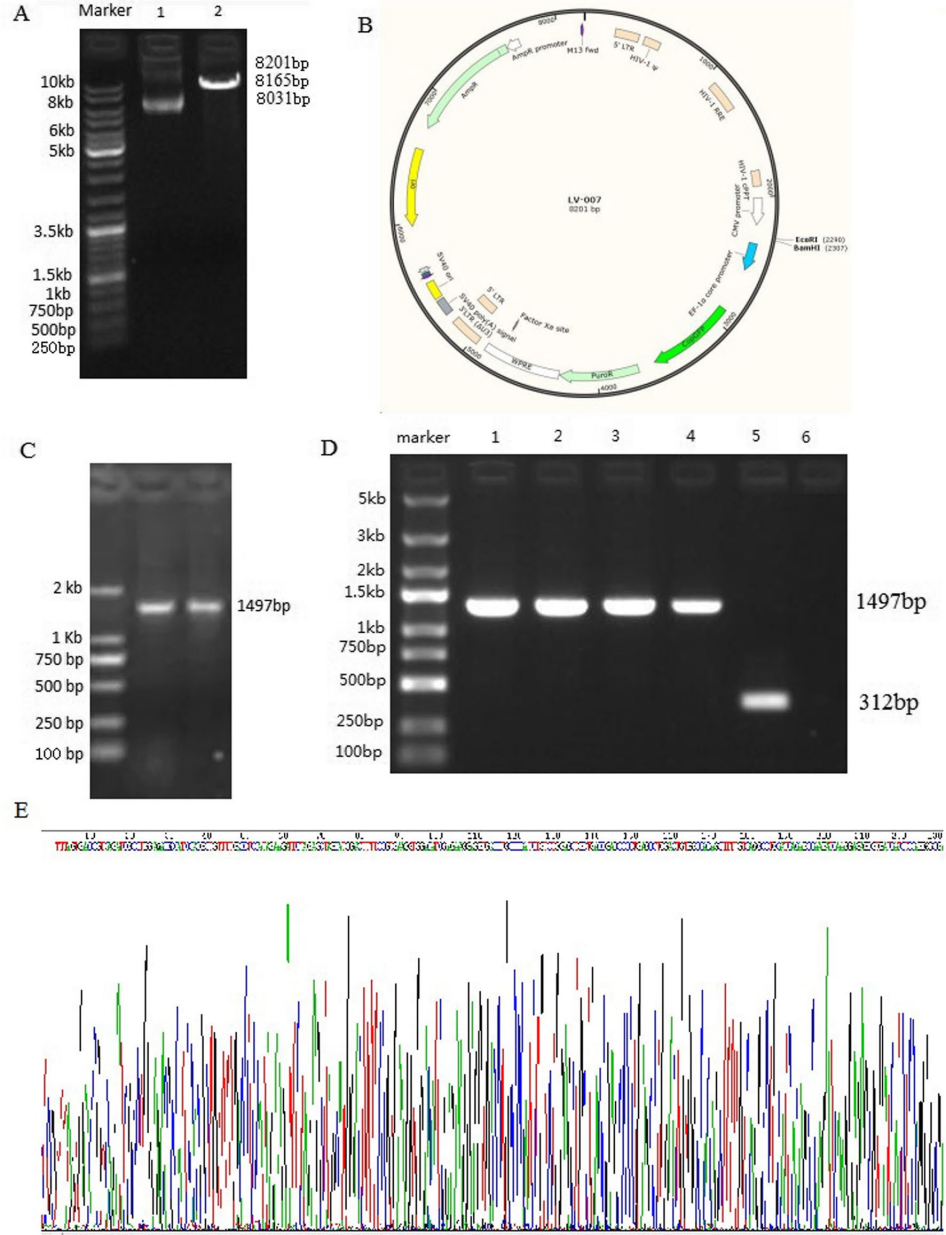
Establishment of a TRIM22 overexpression plasmid. **A** Results of agarose gel electrophoresis of the restriction enzyme-digested vector; 1 LV-007 Vector linearized by Nhe I and Age I double digestion, 2 LV-007 Vector (vector plasmid without digestion); **B** Lv-007 vector map, and Nhe I and Age I enzyme digestion. The component order is CMV-MCS-EF1-copGFP-T2A-puromycin; The fluorescent tags is copGFP. **C** Electrophoresis of the TRIM22 PCR products. A specific band at ~ 1497 bp, which was consistent with the expected size, was noted. **D** 1–4 positive transformants of the TRIM22 overexpression plasmid; 5 negative control (no-load self-connecting control group). The negative control was used to show no false positives in the amplification process using empty vectors without the target gene as templates. The PCR product size of the negative transformant was 312 bp; 6 Negative control (ddH2O). **E** False positives result from the contamination of external nucleic acid in the exclusion system

The vector map of a TRIM22 overexpression plasmid is shown in Fig. 15B. The linearized vector was obtained by Nhe I and Age I digestion, and the target gene fragment (Fig. 15A) was obtained by PCR amplification (Fig. 15C). The homologous recombination sequence was added to the 5′-end of the amplicon, and the 5′- and 3′-terminal sequences of the amplified product matched the linearized clone vector. The linearized vector and target gene fragment were recombined in vitro and transformed in *E. coli*. PCR product identification was done (Fig. 15D), and the results of positive clone sequencing were identical to the expected target sequence (see Appendix).

### Detection of TRIM22 overexpression efficiency

**Fig. 16:**
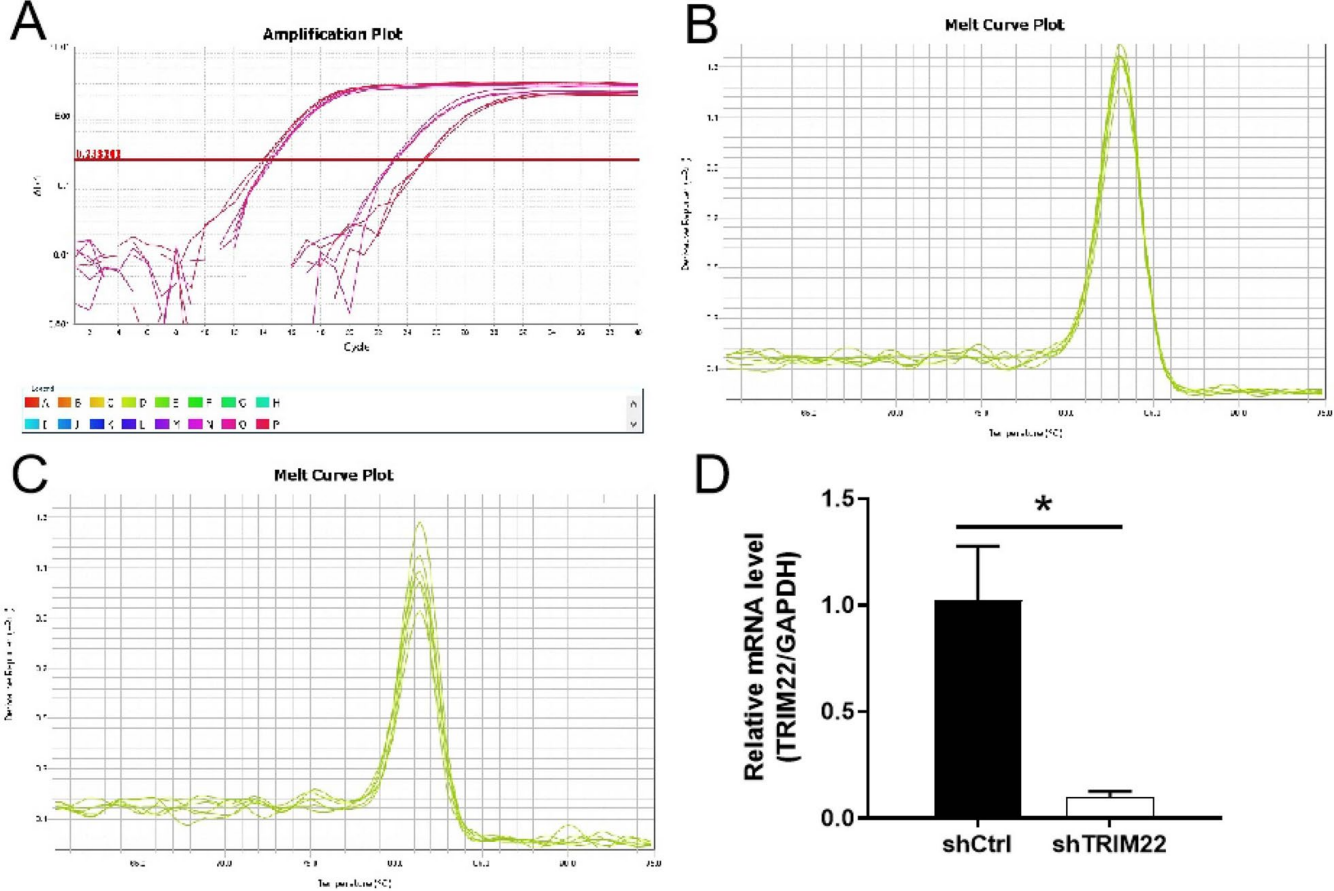
Overexpression efficiency detection. **A** Amplication plot, Letters A–P represent different samples; **B** Melt curve plot of H-GAPDH; **C** Melt curve plot of H-TRIM22; **D** The mRNA expression of TRIM22 after TRIM22 overexpression was detected by RT-qPCR. **P < 0.01

RT-qPCR results showed that compared with the empty vector group, the TRIM22 mRNA expression level in the TRIM22-OE group significantly increased (*P* < 0.01) (Fig. 16A–D).

### Effect of TRIM22 overexpression on autophagy-related proteins following DENV-2-infection of HUVECs

**Fig. 17:**
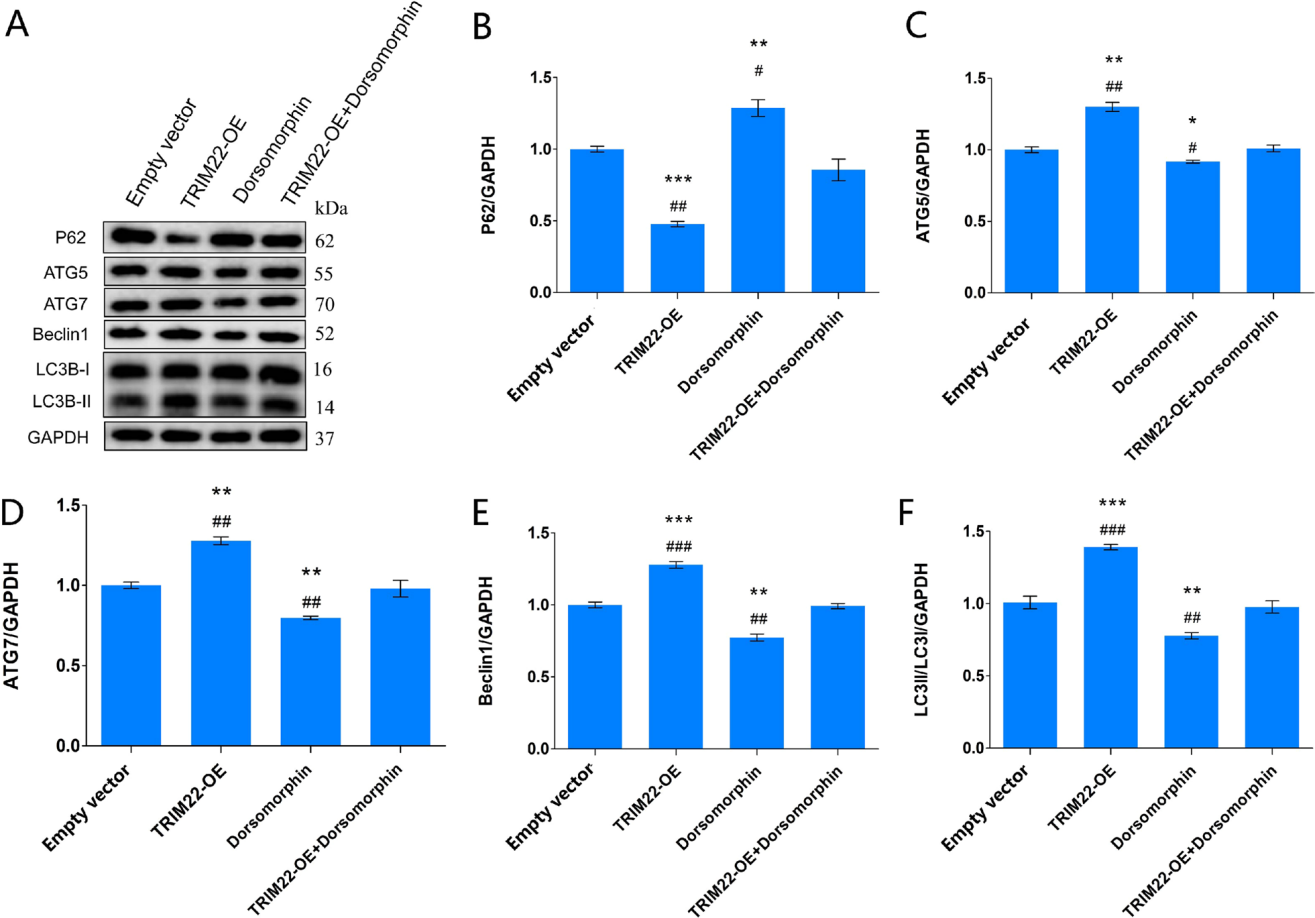
Effect of TRIM22 overexpression on HUVEC autophagy-related factors. **A** The effect of TRIM22 overexpression on autophagy-related factors in DENV-2-infected HUVECs as detected by western blot analysis; **B**–**F** The quantitative expressions analysis autophagy-related proteins. TRIM22-OE: TRIM22 overexpression group; Dorsomorphin: AMPK inhibitor. Empty vector: DENV-2 + Empty vector group; TRIM22-OE: DENV-2 + TRIM22-OE group; 3-MA: DENV-2 + Empty vector + Dorsomorphin; Dorsomorphin + TRIM22-OE: DENV-2 + TRIM22-0E + Dorsomorphin group. *represents comparison with the Empty vector group; *P < 0.05, **P < 0.01, and ***P < 0.001. #represents comparison with the TRIM22-OE + Dorsomorphin group; #*P* < 0.05, ##*P* < 0.01, and ###*P* < 0.001

Compared with the Empty vector group, after DENV-2 infection of TRIM22-overexpressing HUVECs, the expression of ATG5 in the TRIM22-OE group increased (*P* < 0.01); the expression levels of ATG7, Beclin1, and LC3B-II increased (*P* < 0.001); and the expression of P62 decreased (*P* < 0.001). The expression of ATG5 (*P* < 0.05), ATG7 (*P* < 0.001), Beclin1 (*P* < 0.01), and LC3II/LC3I (*P* < 0.05) in the Dorsomorphin group decreased, whereas the P62 expression levels increased (*P* < 0.01). This indicates that AMPK inhibitors inhibit autophagy. Compared with the TRIM22-OE group, the expression of ATG5 (*P* < 0.05), ATG7 (*P* < 0.01), Beclin1 (*P* < 0.01), and LC3II/LC3I (*P* < 0.01) in the TRIM22-OE + Dorsomorphin group decreased, whereas P62 expression levels increased (*P* < 0.05). Compared with the Dorsomorphin group, the expression of ATG5 (*P* < 0.01), ATG7 (*P* < 0.001), Beclin1 (*P* < 0.001), and LC3II/LC3I (*P* < 0.001) in the TRIM22-OE + 3-MA group increased, whereas P62 expression levels decreased (*P* < 0.01) (Fig. 17A–F). These results indicate that TRIM22 reduces the inhibitory effects of AMPK inhibitors on autophagy, suggesting that TRIM22 promotes autophagy induced by DENV-2 infection.

### Effect of TRIM22 overexpression on AMPK-related proteins in DENV-2-infected HUVECs

**Fig. 18:**
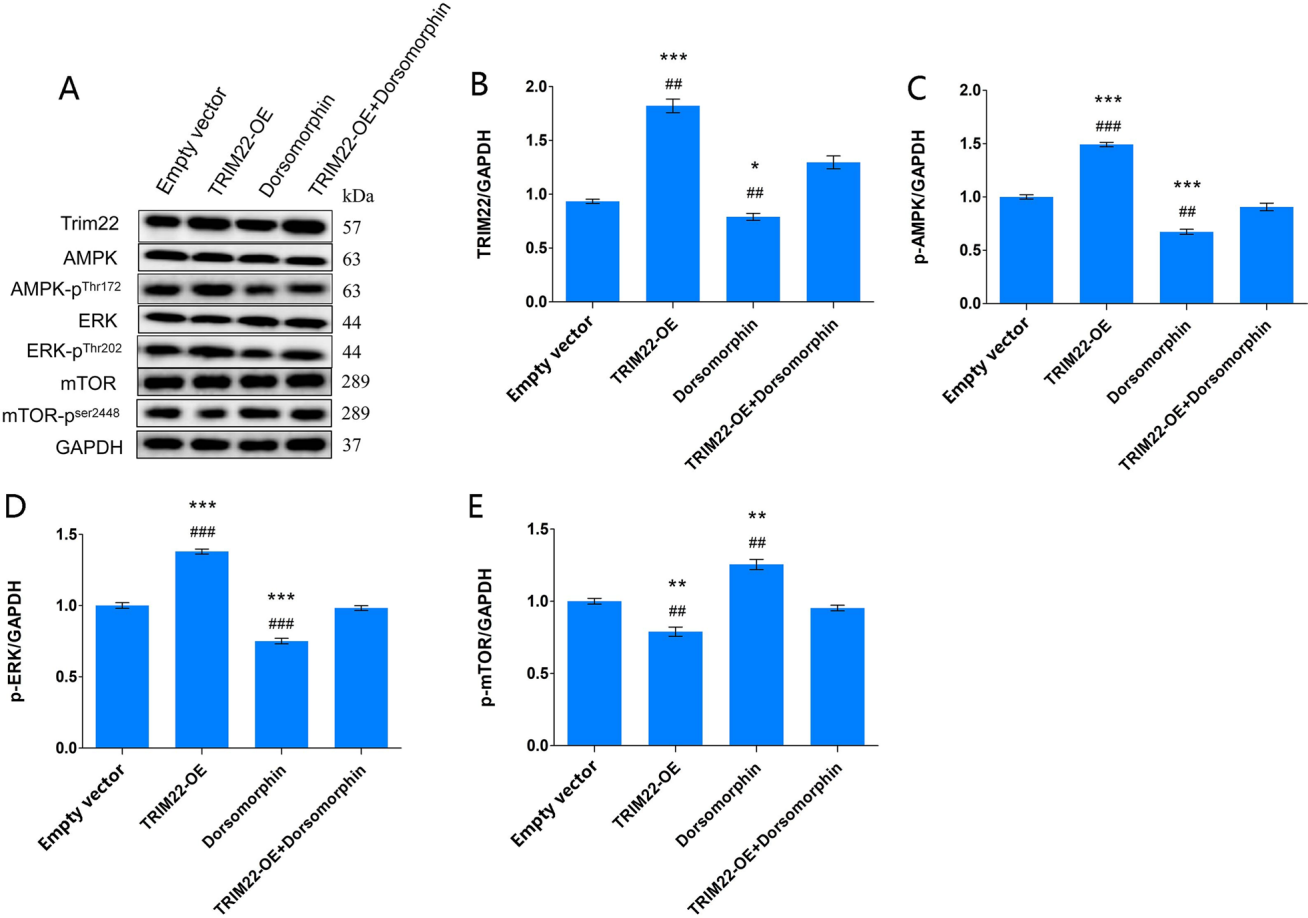
Effects of TRIM22 overexpression on AMPK/ERK/mTOR signaling pathway-related proteins. **A** The effect of TRIM22 overexpression on the expressions of AMPK/ERK/mTOR pathway proteins in DENV-2-infected HUVECs as detected by western blot analysis; **B**–**E** The quantitative expressions analysis of AMPK pathway-related proteins. *represents comparison with the Empty vector group; *P < 0.05, **P < 0.01, and ***P < 0.001. #represents comparison with the TRIM22-OE + Dorsomorphin group, #*P* < 0.05, ##*P* < 0.01, ###*P* < 0.001

Compared with the Empty vector group, after DENV-2 infection of HUVECs with TRIM22 overexpression, the expression of p-AMPK (*P* < 0.001) and p-ERK (*P* < 0.001) in the TRIM22-OE group increased, whereas p-mTOR expression levels decreased (*P* < 0.01). These results indicate that TRIM22 activates the AMPK/ERK/mTOR pathway. In the Dorsomorphin group, the expression levels of p-AMPK (*P* < 0.001) and p-ERK (*P* < 0.001) decreased, whereas p-mTOR expression levels increased (*P* < 0.01). These results indicated that Dorsomorphin inhibits the expression of the AMPK/ERK/mTOR pathway. Compared with the TRIM22-OE group, the expression of p-AMPK (*P* < 0.01) and p-ERK (*P* < 0.01) in the TRIM22-OE + Dorsomorphin group was downregulated, whereas the p-mTOR protein expression levels were upregulated (*P* < 0.01). Compared with the Dorsomorphin group, the expression of p-AMPK (*P* < 0.001) and p-ERK (*P* < 0.001) in the TRIM22-OE + Dorsomorphin group was upregulated, whereas p-mTOR expression levels were downregulated (*P* < 0.01) (Fig. 18A–E). These results indicate that TRIM22 overexpression reduces the inhibitory effect of AMPK inhibitors on the AMPK/ERK/mTOR pathway, suggesting that TRIM22 enhances DENV-2 infection-induced autophagy through the AMPK/ERK/mTOR signaling pathway.

## Discussion

In recent years, studies have suggested that DENV induces autophagy to prevent host cell death and enhance viral replication. Activation of autophagy regulates lipid metabolism and provides materials and energy for DENV replication, whereas inhibition of autophagy results in a significant decrease in viral replication [14, 15]. Previous studies showed that DENV-2-infected HUVECs induced autophagy through the AMPK/ERK/mTOR signaling pathway; however, which host cell molecules are activated by DENV-2 to regulate AMPK and ERK1/2 signaling is unclear. Therefore, the mechanism of DENV-2 activation of the AMPK/ERK/mTOR signaling pathway was examined to induce autophagy at the host protein level.

TRIM22 belongs to the TRIM protein family, which contains ~ 80 members. It is characterized as an N-terminal RING domain, one or two B-box domains, and a curly helix domain. Of these, the RING domain exhibits E3 ubiquitin ligase activity, whereas the B-box and curly helix domain mediate protein–protein interactions [20]. Members of the TRIM family are involved in various biological processes including cell cycle regulation, autophagy, antiviral immunity, tumorigenesis, and tumor progression [21]. TRIM22 expression may be induced by interferon and its 5′ flanking region gene contains two homologous sequences of IFN-stimulated response elements, which bind to IFN regulatory factor 1 in response to types I and II IFN stimulation and induce TRIM22 expression [22]. In addition to interferon, TRIM22 expression is also regulated by viruses and viral antigens [23]. As a member of the TRIM family, TRIM22 regulates viral infection through various mechanisms. Since its discovery, TRIM22 is characterized by its ability to inhibit HIV-1 transcription. TRIM22 inhibits basic HIV-1 transcription by preventing Sp1 from binding to the HIV-1 promoter, thus inhibiting HIV-1 infection [24]. The binding of TRIM22 to the influenza virus nuclear protein promotes its downregulation through ubiquitination degradation and plasmosome dependence to inhibit influenza virus replication [25]. TRIM22 also inhibits herpes simplex viruses by promoting chromatin compression to silence viral DNA encoding early viral genes [26]. TRIM22 also inhibits hepatitis B virus and gamma herpesvirus [27]. Recent studies showed that the TRIM protein family is also involved in DENV infection. TRIM69 degrades Lys104 amino acid residues of DENV NS3 by ubiquitinating its RING domain through E3 ubiquitin ligase activity to inhibit DENV replication [28]. The subgenomic RNA of DENV-2 inhibits RIG-I activation and IFN expression by binding to TRIM25 [29].

Autophagy is a highly conserved intracellular catabolic process and abnormal autophagy is closely associated with the occurrence and development of various diseases [30]. Several studies have demonstrated that TRIM family members can regulate autophagy through various pathways, including the regulation of autophagy-related signaling pathways and autophagy core molecules as autophagy substrate recognition receptors [31]. Related studies have indicated that TRIM5α promotes the initiation of autophagy by promoting the interaction between activated ULK1 and Beclin1 [32]. TRIM39 knockdown enhances the accumulation of autophagosomes and promotes autophagic flux in a Rab7-dependent manner [33]. TRIM65 knockdown inhibits autophagy of A549/DDP cells through the miR-138-5P/ATG7 pathway [34], whereas TRIM14 promotes autophagy in gastric cancer cells by activating the AMPK pathway [35].

Autophagy plays a dual role in viral infection. It inhibits viral replication and can also be exploited by the virus to promote its replication [36]. Viruses that are cleared by autophagy include herpes simplex virus type I, sindbis virus, and human immunodeficiency virus type I. Viruses that utilize autophagy to promote their replication include Coxsackie virus B3, hepatitis C virus, and Zika virus [34, 36]. Similarly, DENV induces autophagy to prevent host cell death and enhance its viral replication. In addition, activation of autophagy regulates lipid metabolism and provides the energy and materials for DENV replication, while inhibition of autophagy leads to a significant decrease in viral replication [11, 14]. During viral infection, the TRIM family regulates virus–host cell interactions by regulating autophagy. Several mechanisms involving TRIM proteins in virus-induced autophagy have been reported. Some TRIM proteins act as specific substrate receptors that directly recognize viral components, which they target to mediate autophagic degradation. Some TRIM proteins regulate the activity of key signaling proteins involved in various steps of the autophagy pathway [37]. TRIM5α not only directly recognizes the HIV capsid protein to mediate its autophagic degradation but also promotes the degradation of the HIV capsid protein by regulating autophagy-related molecules [38]. TRIM16 promotes antiviral autophagy by promoting the activation of the P62-NRF2 axis [39]. TRIM23 mediates virus-induced autophagy by activating TANK-bound kinase 1 (TBK1) [40]. Similar studies indicate that TRIM22 can also regulate autophagy. TRIM22 regulates macrophage autophagy through NF-κB/Beclin1 signaling [17], promotes GEM-induced prosurvival autophagy, and protects NSCLC cells from apoptosis [18]. TRIM22, however, promotes viral replication by regulating autophagy. Related studies have shown that TRIM22 binds to the autophagy-related proteins, ULK1 and Beclin1, to induce autophagy, thus promoting RSV replication [19].

Based on the above findings and previous proteomics results, the current study hypothesized that TRIM22 may be involved in the autophagic regulation of HUVECs infected with DENV-2. The present study first determined the virulence of DENV-2 and the optimal concentration required to induce HUVEC autophagy. TRIM22 expression was increased in DENV-2-infected HUVECs, confirming the previous proteomic results. TRIM22 was also knocked down in HUVECs causing the proliferation rate to decrease, the expression of autophagy-related proteins to decrease, and autolysosomes to increase. This suggests that TRIM22 knockdown inhibits autophagy, which is consistent with previous reports that TRIM22 promotes autophagy. Moreover, following TRIM22 knockdown, protein phosphorylation levels of AMPK and ERK decreased, whereas that of mTOR increased. Previous studies showed that AMPK/ERK/mTOR signaling pathway plays a key role in the regulation of autophagy, and its main mechanism is that AMPK regulates autophagy by inhibiting downstream mTOR, which may be an activation mode of AMPK/ERK/mTOR signal in the regulation of autophagy [41–43]. These results suggest that TRIM22 activates autophagy through the AMPK/ERK/mTOR signaling pathway. TRIM22 induces autophagy in HUVECs through the AMPK/ERK/mTOR signaling pathway.

To determine whether TRIM22 plays a role in regulating autophagy following DENV-2 infection of HUVECs, the current study examined DENV-2-infected HUVECs with TRIM22 knockdown. HUVECs in the G2 phase of the cell cycle increased, apoptosis increased, autophagy-related protein levels decreased, and the number of autolysosomes increased. This indicates that an effect on TRIM22 on HUVEC autophagy occurs during DENV-2 infection. Moreover, TRIM22 knockdown during DENV-2 infection ameliorates the activation effect of an autophagy activator on HUVEC autophagy. These results suggest that TRIM22 promotes autophagy induced by HUVECs infected with DENV-2. Autophagy and apoptosis control intracellular homeostasis. In general, autophagy blocks the induction of apoptosis, enabling cells to adapt to environmental stress. Both processes are under the control of multiple common upstream signals, and cross-regulation exists between them as a form of inhibition [44]. Therefore, TRIM22 knockdown results in increased HUVECs in the G2 phase of the cell cycle and increased apoptosis may be caused by autophagy inhibition. To confirm the regulation of TRIM22 on autophagy, TRIM22 was overexpressed in HUVECs and infected with DENV-2. The results indicated that autophagy increased and TRIM22 overexpression reduced the inhibitory effect of autophagy inhibitors on autophagy. The increased expression of TRIM22 during DENV-2 infection was confirmed to promote HUVEC autophagy. TRIM22 was knocked down and overexpressed in HUVECs followed by infection with DENV-2 to further explore whether TRIM22 mediates DENV-2-induced autophagy through the AMPK/ERK/mTOR pathway. Following TRIM22 knockdown, protein phosphorylation levels of AMPK and ERK decreased, whereas that of mTOR increased. In addition, the positive regulatory effect of an autophagy activator on the AMPK/ERK/mTOR pathway decreased by TRIM22 knockdown. In contrast, activation of the AMPK/ERK/mTOR pathway increased after TRIM22 overexpression, and the negative regulatory effect of autophagy inhibitors on the AMPK/ERK/mTOR pathway was reduced as a result of TRIM22 overexpression. This indicates that TRIM22 is involved in the autophagy activation of DENV-2-infected HUVECs through the AMPK/ERK/mTOR pathway.

This study confirmed, for the first time, that TRIM22 is involved in DENV-2-induced autophagy through the AMPK/ERK/mTOR signaling pathway; however, the details of this regulatory mechanism remain to be defined. Future studies will explore the mechanism through which DENV-2 promotes TRIM22 expression and the relationship between TRIM22 and the AMPK pathway to better understand the mechanism of DENV-2-induced autophagy.

## Supplementary Information

**Additional file 1.** Original data of Western Blot results.

## Data Availability

The datasets used and/or analysed during the current study are available from the corresponding author on reasonable request.

## Abbreviations

DENV-2: Dengue virus type 2
HUVECs: Primary human umbilical vein endothelial cells
AMPK: Adenosine monophosphate-activated protein kinase
ERK: Extracellular signal-regulated kinase
mTOR: Mammalian target of rapamycin
TRIM22: Tripartite motif-containing 22
qRT-PCR: Quantitative real-time polymerase chain reaction
TEM: Transmission electron microscopy
FCM: Flow cytometry
ATG7: Antithymocyte globulin 7
ATG5: Antithymocyte globulin 5
DENV: Dengue virus
DF: Dengue fever
DHF: Dengue hemorrhagic fever
DSS: Dengue shock syndrome
TSC2: Tuberous sclerosis complex 2
NSCLC: Non-small cell lung cancer
ULK1: Unc-51-like autophagy activating kinase 1
RSV: Respiratory syncytial virus
STRING: Search tool for the retrieval of interacting genes/proteins
ECM: Extracellular matrix
PCR: Polymerase chain reaction
LR: Early apoptotic cell
UR: Late apoptotic cell
OE: Overexpression group
AICAR: 5-aminoimidazole-4-carboxamide 1-β-D-ribofuranoside
TBK1: TANK-bound kinase 1
