# Supplementary figures and images for: Mechanism of autophagy induced by activation of the AMPK/ERK/mTOR signaling pathway after TRIM22-mediated DENV-2 infection of HUVECs
Source: Virol J. 2022 Dec 31;19:228. doi: 10.1186/s12985-022-01932-w (PMC9805691; doi:10.1186/s12985-022-01932-w)

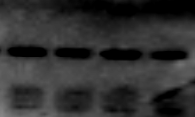

Supplement: Supplementary file 1 — Additional file 1. Original data of Western Blot results. [file 12985_2022_1932_MOESM1_ESM.zip › Additional documents/Effects of TRIM22 knockdown on DENV-2-induced autophagy and AMPK pathway-related protein expression/AMPK.png]

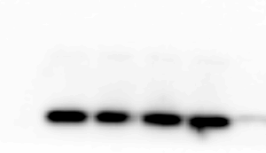

Supplement: Supplementary file 1 — Additional file 1. Original data of Western Blot results. [file 12985_2022_1932_MOESM1_ESM.zip › Additional documents/Effects of TRIM22 knockdown on DENV-2-induced autophagy and AMPK pathway-related protein expression/ATG1.png]

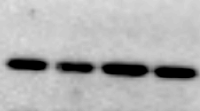

Supplement: Supplementary file 1 — Additional file 1. Original data of Western Blot results. [file 12985_2022_1932_MOESM1_ESM.zip › Additional documents/Effects of TRIM22 knockdown on DENV-2-induced autophagy and AMPK pathway-related protein expression/ATG5.png]

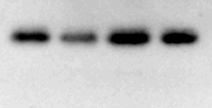

Supplement: Supplementary file 1 — Additional file 1. Original data of Western Blot results. [file 12985_2022_1932_MOESM1_ESM.zip › Additional documents/Effects of TRIM22 knockdown on DENV-2-induced autophagy and AMPK pathway-related protein expression/ATG7.png]

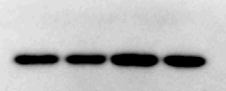

Supplement: Supplementary file 1 — Additional file 1. Original data of Western Blot results. [file 12985_2022_1932_MOESM1_ESM.zip › Additional documents/Effects of TRIM22 knockdown on DENV-2-induced autophagy and AMPK pathway-related protein expression/Beclin1.png]

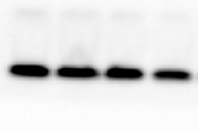

Supplement: Supplementary file 1 — Additional file 1. Original data of Western Blot results. [file 12985_2022_1932_MOESM1_ESM.zip › Additional documents/Effects of TRIM22 knockdown on DENV-2-induced autophagy and AMPK pathway-related protein expression/ERK.png]

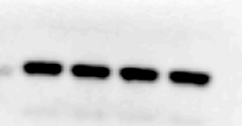

Supplement: Supplementary file 1 — Additional file 1. Original data of Western Blot results. [file 12985_2022_1932_MOESM1_ESM.zip › Additional documents/Effects of TRIM22 knockdown on DENV-2-induced autophagy and AMPK pathway-related protein expression/GAPDH.png]

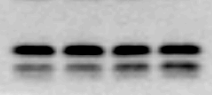

Supplement: Supplementary file 1 — Additional file 1. Original data of Western Blot results. [file 12985_2022_1932_MOESM1_ESM.zip › Additional documents/Effects of TRIM22 knockdown on DENV-2-induced autophagy and AMPK pathway-related protein expression/LC3B.png]

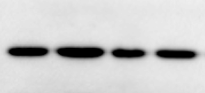

Supplement: Supplementary file 1 — Additional file 1. Original data of Western Blot results. [file 12985_2022_1932_MOESM1_ESM.zip › Additional documents/Effects of TRIM22 knockdown on DENV-2-induced autophagy and AMPK pathway-related protein expression/P62.png]

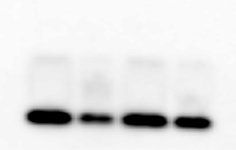

Supplement: Supplementary file 1 — Additional file 1. Original data of Western Blot results. [file 12985_2022_1932_MOESM1_ESM.zip › Additional documents/Effects of TRIM22 knockdown on DENV-2-induced autophagy and AMPK pathway-related protein expression/TRIM22.png]

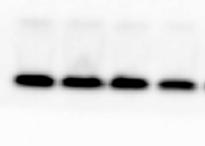

Supplement: Supplementary file 1 — Additional file 1. Original data of Western Blot results. [file 12985_2022_1932_MOESM1_ESM.zip › Additional documents/Effects of TRIM22 knockdown on DENV-2-induced autophagy and AMPK pathway-related protein expression/mTOR.png]

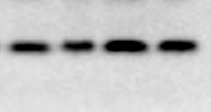

Supplement: Supplementary file 1 — Additional file 1. Original data of Western Blot results. [file 12985_2022_1932_MOESM1_ESM.zip › Additional documents/Effects of TRIM22 knockdown on DENV-2-induced autophagy and AMPK pathway-related protein expression/p-AMPK.png]

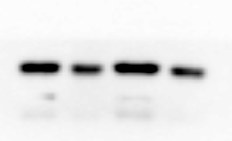

Supplement: Supplementary file 1 — Additional file 1. Original data of Western Blot results. [file 12985_2022_1932_MOESM1_ESM.zip › Additional documents/Effects of TRIM22 knockdown on DENV-2-induced autophagy and AMPK pathway-related protein expression/p-ERK.png]

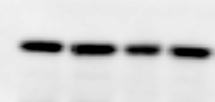

Supplement: Supplementary file 1 — Additional file 1. Original data of Western Blot results. [file 12985_2022_1932_MOESM1_ESM.zip › Additional documents/Effects of TRIM22 knockdown on DENV-2-induced autophagy and AMPK pathway-related protein expression/p-mTOR.png]

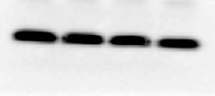

Supplement: Supplementary file 1 — Additional file 1. Original data of Western Blot results. [file 12985_2022_1932_MOESM1_ESM.zip › Additional documents/Effects of TRIM22 overexpression on DENV-2-infected HUVEC autophagy and AMPK pathway-related proteins/AMPK.png]

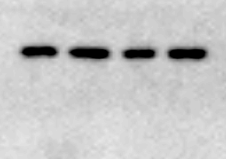

Supplement: Supplementary file 1 — Additional file 1. Original data of Western Blot results. [file 12985_2022_1932_MOESM1_ESM.zip › Additional documents/Effects of TRIM22 overexpression on DENV-2-infected HUVEC autophagy and AMPK pathway-related proteins/ATG5.png]

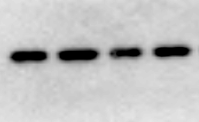

Supplement: Supplementary file 1 — Additional file 1. Original data of Western Blot results. [file 12985_2022_1932_MOESM1_ESM.zip › Additional documents/Effects of TRIM22 overexpression on DENV-2-infected HUVEC autophagy and AMPK pathway-related proteins/ATG7.png]

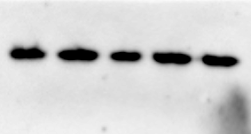

Supplement: Supplementary file 1 — Additional file 1. Original data of Western Blot results. [file 12985_2022_1932_MOESM1_ESM.zip › Additional documents/Effects of TRIM22 overexpression on DENV-2-infected HUVEC autophagy and AMPK pathway-related proteins/Beclin1.png]

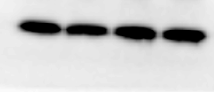

Supplement: Supplementary file 1 — Additional file 1. Original data of Western Blot results. [file 12985_2022_1932_MOESM1_ESM.zip › Additional documents/Effects of TRIM22 overexpression on DENV-2-infected HUVEC autophagy and AMPK pathway-related proteins/ERK.png]

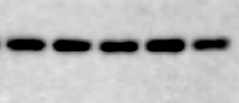

Supplement: Supplementary file 1 — Additional file 1. Original data of Western Blot results. [file 12985_2022_1932_MOESM1_ESM.zip › Additional documents/Effects of TRIM22 overexpression on DENV-2-infected HUVEC autophagy and AMPK pathway-related proteins/GAPDH.png]

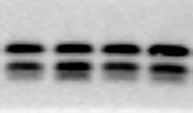

Supplement: Supplementary file 1 — Additional file 1. Original data of Western Blot results. [file 12985_2022_1932_MOESM1_ESM.zip › Additional documents/Effects of TRIM22 overexpression on DENV-2-infected HUVEC autophagy and AMPK pathway-related proteins/LC3B.png]

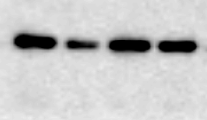

Supplement: Supplementary file 1 — Additional file 1. Original data of Western Blot results. [file 12985_2022_1932_MOESM1_ESM.zip › Additional documents/Effects of TRIM22 overexpression on DENV-2-infected HUVEC autophagy and AMPK pathway-related proteins/P62.png]

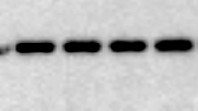

Supplement: Supplementary file 1 — Additional file 1. Original data of Western Blot results. [file 12985_2022_1932_MOESM1_ESM.zip › Additional documents/Effects of TRIM22 overexpression on DENV-2-infected HUVEC autophagy and AMPK pathway-related proteins/mTOR.png]

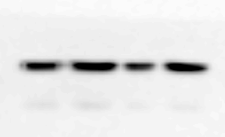

Supplement: Supplementary file 1 — Additional file 1. Original data of Western Blot results. [file 12985_2022_1932_MOESM1_ESM.zip › Additional documents/Effects of TRIM22 overexpression on DENV-2-infected HUVEC autophagy and AMPK pathway-related proteins/p-AMPK.png]

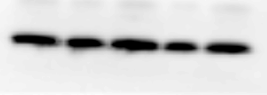

Supplement: Supplementary file 1 — Additional file 1. Original data of Western Blot results. [file 12985_2022_1932_MOESM1_ESM.zip › Additional documents/Effects of TRIM22 overexpression on DENV-2-infected HUVEC autophagy and AMPK pathway-related proteins/p-ERK.png]

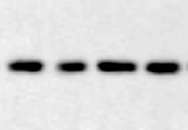

Supplement: Supplementary file 1 — Additional file 1. Original data of Western Blot results. [file 12985_2022_1932_MOESM1_ESM.zip › Additional documents/Effects of TRIM22 overexpression on DENV-2-infected HUVEC autophagy and AMPK pathway-related proteins/p-mTOR.png]

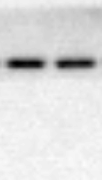

Supplement: Supplementary file 1 — Additional file 1. Original data of Western Blot results. [file 12985_2022_1932_MOESM1_ESM.zip › Additional documents/TRIM22 knockdown affects the expression of autophagy-related proteins in HUVECs/AMPK.png]

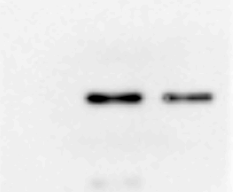

Supplement: Supplementary file 1 — Additional file 1. Original data of Western Blot results. [file 12985_2022_1932_MOESM1_ESM.zip › Additional documents/TRIM22 knockdown affects the expression of autophagy-related proteins in HUVECs/ATG5.png]

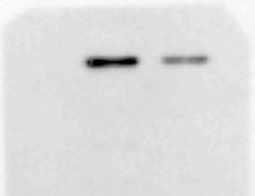

Supplement: Supplementary file 1 — Additional file 1. Original data of Western Blot results. [file 12985_2022_1932_MOESM1_ESM.zip › Additional documents/TRIM22 knockdown affects the expression of autophagy-related proteins in HUVECs/ATG7.png]

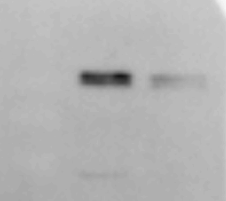

Supplement: Supplementary file 1 — Additional file 1. Original data of Western Blot results. [file 12985_2022_1932_MOESM1_ESM.zip › Additional documents/TRIM22 knockdown affects the expression of autophagy-related proteins in HUVECs/Beclin1.png]

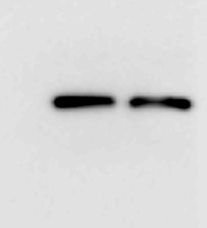

Supplement: Supplementary file 1 — Additional file 1. Original data of Western Blot results. [file 12985_2022_1932_MOESM1_ESM.zip › Additional documents/TRIM22 knockdown affects the expression of autophagy-related proteins in HUVECs/ERK.png]

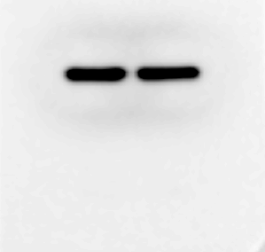

Supplement: Supplementary file 1 — Additional file 1. Original data of Western Blot results. [file 12985_2022_1932_MOESM1_ESM.zip › Additional documents/TRIM22 knockdown affects the expression of autophagy-related proteins in HUVECs/GAPDH.png]

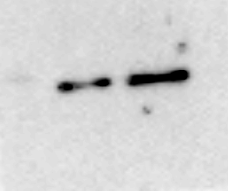

Supplement: Supplementary file 1 — Additional file 1. Original data of Western Blot results. [file 12985_2022_1932_MOESM1_ESM.zip › Additional documents/TRIM22 knockdown affects the expression of autophagy-related proteins in HUVECs/P62.png]

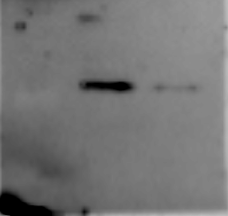

Supplement: Supplementary file 1 — Additional file 1. Original data of Western Blot results. [file 12985_2022_1932_MOESM1_ESM.zip › Additional documents/TRIM22 knockdown affects the expression of autophagy-related proteins in HUVECs/TRIM22.png]

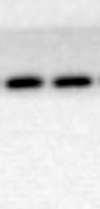

Supplement: Supplementary file 1 — Additional file 1. Original data of Western Blot results. [file 12985_2022_1932_MOESM1_ESM.zip › Additional documents/TRIM22 knockdown affects the expression of autophagy-related proteins in HUVECs/mTOR.png]

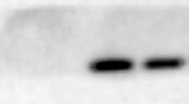

Supplement: Supplementary file 1 — Additional file 1. Original data of Western Blot results. [file 12985_2022_1932_MOESM1_ESM.zip › Additional documents/TRIM22 knockdown affects the expression of autophagy-related proteins in HUVECs/p-AMPK.png]

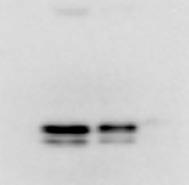

Supplement: Supplementary file 1 — Additional file 1. Original data of Western Blot results. [file 12985_2022_1932_MOESM1_ESM.zip › Additional documents/TRIM22 knockdown affects the expression of autophagy-related proteins in HUVECs/p-ERK.png]
